# Timing matters: early administration of a high-affinity antibody targeting the tau repeat domain prevents aggregation in a mouse tauopathy model

**DOI:** 10.1186/s13195-026-01985-x

**Published:** 2026-02-14

**Authors:** Lewis K. Penny, Mohammad Arastoo, Richard Lofthouse, Aya Abdallah, Peter I. Imoesi, Karima Schwab, Helen Shiells, Valeria Melis, Gernot Riedel, Charles R. Harrington, Claude M. Wischik, Andrew Porter, Soumya Palliyil

**Affiliations:** 1 Institute of Medical Sciences, University of Aberdeen, Aberdeen, UK; 2 Scottish Biologics Facility, University of Aberdeen, Aberdeen, UK; 3 GT Diagnostics (UK) Ltd., Aberdeen, UK; 4 TauRx Therapeutics Management Ltd., Aberdeen, UK

**Keywords:** Alzheimer’s disease, Tauopathy, Tau protein, Paired helical filament, Monoclonal antibody, Diagnostic, Therapy, Immunotherapy, S1D12

## Abstract

**Introduction:**

Immunotherapy is an attractive proposition for preventing the spread of pathologic tau in Alzheimer’s disease and other tauopathies. Given that tau is heavily truncated in tauopathies, it is hypothesised that directly targeting the repeat domain which forms the core of pathological filaments will improve the likelihood of success. S1D12, a chimeric IgG2a isolated via phage display, recognises 2N4R tau341-353 with high affinity (200 pM) and has previously been shown to prevent tau aggregation and propagation in vitro. We further explored the pharmacokinetics and biodistribution of S1D12 as well as its efficacy in a tauopathy mouse model. We also verified its efficacy in vitro against tau seeding species from multiple human tauopathies.

**Methods:**

Single dose S1D12 intraperitoneal injections (30 mg/kg) were performed in wild-type mice followed by tissue harvest at multiple time points. For efficacy studies, four-weekly doses followed by four-fortnightly doses of S1D12 (30 mg/kg) or negative control antibody were administered intraperitoneally to Line 66 tau transgenic mice. Two cohorts, beginning from 2 months and 4.5 months of age were utilised. Endpoints included quantification of aggregated tau, seed-competent tau and insoluble phosphorylated tau in brain homogenates, as well as neurofilament light (NfL), tau phosphorylated at Thr-217 (pTau217) and core tau in plasma.

**Results:**

S1D12 was detected in plasma and in brain with a t_max_ of 24/48 h respectively, and a slow washout over 7 days (t_1/2_ > 230 h), with 0.35% CNS bioavailability. S1D12 inhibited the generation of aggregated tau, seed-competent tau and insoluble phosphorylated tau in transgenic mice. This was associated with a reduction in NfL and pTau217, and an increase in core tau in plasma. In the 4.5-month cohort, S1D12 did not remove already established tau aggregates below baseline. Additionally, S1D12 inhibited seeding tau species from different tauopathies to a similar degree, independent of structural diversity.

**Conclusions:**

S1D12, a high-affinity antibody targeting the R4 repeat domain of tau offers potential for halting progression of tau pathology through inhibition of tau aggregation rather than removal of established aggregates. These findings support the notion that both early diagnosis and intervention are key for the treatment of AD and other tauopathies.

**Supplementary Information:**

The online version contains supplementary material available at 10.1186/s13195-026-01985-x.

## Introduction

After decades of effort and attrition in Alzheimer’s disease (AD) clinical trials [1, 2], the first wave of disease-modifying therapies has emerged. Aducanumab [3], lecanemab [4] and donanemab [5] are amyloid beta (Aβ)-targeting monoclonal antibodies (mAbs) which have been approved by the FDA for treating patients with mild cognitive impairment or early symptomatic stages of AD.

Despite these approvals, the modest reduction in clinical decline has led to debate over the real-life efficacy of these drugs [6], whilst adverse effects, including cerebral haemorrhage, oedema, and infusion-related reactions, are common and limiting [4]. In the light of these findings, focus has turned to developing therapies targeting the other major hallmark of the disease, namely tau pathology.

Tau-targeting therapies are considered an attractive disease-modifying approach in AD due to the central role that tau plays in the pathophysiology of the disease [7], and the close correlation between tau pathology and cognitive decline [8, 9]. In the near future, monitoring the spread of tau by brain imaging and the use of tau-specific, fluid biomarkers for measuring tau pathology may be transformational for the diagnosis of AD, triaging of patients for clinical trials, early intervention therapies and monitoring the effect of disease-modifying treatments [5, 10]. Furthermore, tau-targeting therapies could have broad utility across other tauopathies for which there are no approved treatments, e.g., progressive supranuclear palsy, corticobasal degeneration, argyrophilic grain disease, chronic traumatic encephalopathy and frontotemporal dementia [11].

However, anti-tau immunotherapies have not proved successful, and this may be partly explained by the specific tau epitopes that have been targeted by these investigational drugs [12]. Substantial truncation of the tau protein [13, 14], which occurs with aging, is also likely to account for the negative outcomes from these trials. First generation anti-tau immunotherapies have targeted the N- or C-terminal regions of tau rather than the central domain (tau297-391 of the 2N4R tau isoform) that forms the core of the paired helical filaments characteristic of AD brain pathology [15, 16]. Such negative outcomes arose despite clear evidence of mAb engagement with N- and C-termini of tau. For example, gosuranemab, that recognises tau15-22 [17], decreased levels of N-terminal tau in CSF by 98% (compared with 11% increase for placebo) yet demonstrated no clinical efficacy in a progressive supranuclear palsy trial [18]. Similarly, in tau transgenic mice, gosuranemab decreased N-terminal tau in CSF while levels of the mid-region tau remained unaltered [17].

To evaluate an anti-tau immunotherapeutic approach that targets the disease-relevant core domain of tau, we have developed S1D12, a sheep-mouse chimeric IgG2a mAb originally isolated via phage display from an immunised sheep antibody fragment library. S1D12 targets the disease-causing PHF-core domain of tau [19]. It recognises an epitope within tau341-353 with high affinity (200 pM) and has previously been shown to prevent tau aggregation and propagation in biochemical and cellular assays, respectively [19]. For this proof-of-concept study, we have investigated whether S1D12 can affect tau aggregation in vivo in tau transgenic mice.

## Materials and methods

### Test articles—S1D12 and negative control mAbs

S1D12 is a chimeric IgG2a isolated via phage display from an immunised sheep antibody library that targets the disease-causing core of tau [19]. S1D12 contains ovine antibody variable regions and murine IgG2a constant regions. It recognises an epitope within tau341-353 and binds truncated core tau with high affinity (200 pM). In this article, the numbering of tau residues corresponds to the amino acid numbers of the 2N4R isoform of tau. The isotype control mAb B11, also a chimeric antibody with murine IgG2a, binds an irrelevant target.

Large-scale production of both S1D12 and B11 mAbs was performed using a transient expression system in CHO cells and antibody purified through protein A affinity chromatography (mAb production by Evitria AG Switzerland). The yield of purified S1D12 (530 mg/L) was determined by absorption at 280 nm. Purity and monomericity were determined to be 96.7% via analytical size exclusion chromatography (Agilent AdvanceBio SEC column (300A 2.7 µm 7.8 × 300 mm) and PBS as running buffer at 0.8 mL/min.). Endotoxin content of S1D12 was measured using Charles River’s Endosafe PTS system and deemed < 1 EU/mg. For B11 mAb, the purity was 96.9% monomer and endotoxin levels, < 1 EU/mg.

### Mice

Female heterozygous tau-transgenic L66 (*n* = 60) and wild-type NMRI mice (*n* = 42) were commercially bred as previously described [20, 21] in positive-pressure isolators (Charles River, Manston, UK) and delivered to the experimental facilities at the University of Aberdeen, Scotland, by truck one month before testing. They were group-housed in open housing (Tecniplast Type III, 382 × 220 mm, up to 5 mice per cage) with corn cob bedding, paper wool, and cardboard tubes as enrichment. Holding rooms were maintained at a temperature of 20–22 °C, 50–65% humidity, an air exchange rate of 17–20 changes per hour, and a 12-h light/dark cycle with lights turned on at 7 am. Cages were changed once per week and mice had ad libitum access to food and water. The body weights were recorded three times per week during the in-life phase of the studies and averages of the three days were used to calculate a weekly mean. Following animal welfare recommendations any mouse with a body weight loss > 15% was euthanised and excluded from the experiments. No other exclusion criteria applied.

L66 mice overexpress the largest human CNS htau40 isoform (2N4R; 441 amino acid residues), carrying two aggregation-promoting mutations, P301S and G335D. Overexpression was achieved under the control of the neurone-specific mouse Thy1 regulatory element. L66 mice exhibit extensive tau aggregation that leads to frontotemporal dementia (FTD)-like motor dysfunction, and both sexes exhibit similar behavioural and pathological phenotypes [21–24]. Females were selected here in line with our previous work.

Experiments were performed in accordance with the European Communities Council Directive (63/2010/EU) with local ethical approval under the UK Animals (Scientific Procedures) Act (1986) and its Amended Regulations (2012) under the project licence number PP2213334 and complied with the ARRIVE guidelines 2.0 [25].

### Biodistribution of S1D12 in mouse tissue and plasma in a single-dose study

A single intraperitoneal (i.p.) dosing study was conducted to assess the biodistribution of S1D12 mAb using wild-type NMRI mice. Animals were randomly assigned to groups, but no blinding was implemented.

A total of 42 female mice, aged 6 months, were randomly allocated into seven groups (*n* = 6) with 36 mice receiving 30 mg/kg S1D12 and one group receiving vehicle (PBS). Mice were sacrificed 1, 2, 3, 7, 14 or 31 days after dosing to collect blood, whole brain and peripheral tissue samples (liver (right lobe), spleen, kidney (right kidney), muscle (thigh), heart and lung (right inferior lobe)). A summary of the study design is shown in Table 1.

**Table 1:** Details of mice used in single dose S1D12 study

Number of mice	Genotype	Gender	Dose route	Single dose (S1D12) (mg/kg)	Dose (mL/kg)	Sample time point post-dose (h)
6	NMRI	F	i.p	30	5	24
6	NMRI	F	i.p	30	5	48
6	NMRI	F	i.p	30	5	72
6	NMRI	F	i.p	30	5	168
6	NMRI	F	i.p	30	5	336
6	NMRI	F	i.p	30	5	744
6	NMRI	F	i.p	Vehicle (PBS)	5	N/A

*F* Female, *i.p.* Intraperitoneal, *PBS* Phosphate buffered saline, *N/A* Not applicable

Before dosing, bodyweights were recorded and dosing volume (5 mL/kg) calculated. Animals were inspected for clinical signs at dosing and cage-side observations were regular during each week.

At each time point, mice were anaesthetised using an overdose of sodium pentobarbital (i.p.) and blood was collected via cardiac puncture through a Plastipak syringe pre-rinsed with heparinised saline (10 U/mL) and transferred into a plastic vial containing lithium heparin anticoagulant. Blood samples were centrifuged to obtain plasma at 2,000 × g for 5 min at 6 °C. After blood collection, each mouse was perfused with heparinised saline for 2–3 min prior to tissue removal. Samples were snap frozen in liquid nitrogen and stored at −80 °C prior to analysis.

For peripheral tissue biodistribution studies, brain and tissue homogenates were prepared in 1:10 (w/v) RIPA buffer (Cell Signalling, #9806) with 1 × Halt Protease and Phosphatase Inhibitor Cocktail (Thermo Scientific, #78442). Homogenisation was performed with 30 strokes using a Caframo Ultra Torque (BDC1850) with pestle attachment at 200 rpm. Samples were left on ice for up to 1 h and subsequently centrifuged for 10 min at 10,000 × g at 4 °C. The supernatant was collected, and the total protein was quantified using a BCA Protein Assay Kit (Pierce, #23225).

For liver (right lobe), spleen, kidney (right kidney), muscle (thigh), heart and lung (right inferior lobe) tissues, the analysis was performed using samples collected after 168 h. In the case of brain, this was performed for all timepoints with pharmacokinetics properties analysed as described below.

S1D12 concentrations were measured using a capture ELISA method. In brief, a black Nunc MaxiSorp flat-bottom 96-well plate was coated with recombinant dGAE (tau297-391 at 10 µg/mL) and blocked with 2% (w/v) dried milk powder in PBS. S1D12 mAb standard (5 nM) was serially diluted 2-fold across the plate. Tissue homogenates were added to the plate in duplicate wells (2.5% w/v) and the plates were incubated for 1 h at 37 °C. HRP-conjugated anti-mouse IgG (1:1,000 in 2% (w/v) dried milk powder) was added as the secondary antibody and the reaction was developed using SuperSignal ELISA Femto Substrate Solution (Thermo Scientific, #37074) with luminescence read within 5 min of addition. ClarioStar data analysis software (MARS) was used to generate the standard curve (4-parameter non-linear regression) to calculate S1D12 concentrations in various tissues.

For S1D12 levels in plasma, dGAE (1 µg/mL) binding was measured as above by adding doubling dilutions of plasma samples in duplicate from various time points starting with a 1:5,000 dilution. The biodistribution in tissues was reported as free S1D2 mAb concentration (nM) and also expressed as a percentage of the mAb concentration in plasma.

#### Pharmacokinetic (PK) data analysis

PK Solver (Version 2.0) for Microsoft excel [26] was used for PK data analysis. Non-compartmental analysis with extravascular input was performed with a log-linear trapezoidal method. Maximum observed concentration (C_max_), time point of maximum observed concentration (T_max_) and terminal half-life (T_1/2_) of S1D12 were calculated. T_1/2_ was calculated as the natural log of 2 divided by lambda-z where lambda-z is the terminal elimination rate constant calculated from the final three time points measured.

### Proof-of-concept efficacy study – 12-week repeat-dose study

**Fig. 1:**
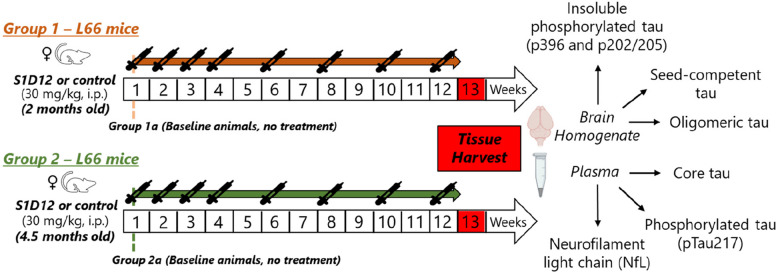
Proof-of-concept efficacy study design – a 12-week repeat-dose study. Four weekly doses followed by four fortnightly doses of S1D12 (30 mg/kg) or negative control antibody (*n* = 10 per group) were administered via intraperitoneal injection to female L66 mice. Mice were sacrificed one week after their final dose. Dosing was performed on two cohorts of mice starting from 2 months and 4.5 months of age (Groups 1 and 2, respectively), with animals sacrificed prior to mAb dosing for surrogate baseline measurements (Groups 1a and 2a, respectively)

Four weekly doses followed by four fortnightly doses of S1D12 (30 mg/kg) or isotype control (*n* = 10 per group) were administered via intraperitoneal injection in female L66 mice. No blinding was applied, but random assignments to groups were implemented and mice sacrificed one week after the final dose. Dosing was performed on two cohorts of mice starting from 2 months and 4.5 months of age (Groups 1 and 2, respectively), with animals sacrificed pre-dosing for surrogate baseline measurements (Groups 1a and 2a, respectively). The study design is summarised in Fig. 1 and cohort details in the supplementary (Table S1). Change in bodyweights were recorded (Supplementary Figures S1 and S2), and dosing volume (5 mL/kg) calculated prior to each dose. Due to the onset of clinical signs, the negative control group received the isotype control mAb until week 8 and vehicle thereafter. Two mice in Group 2 receiving the isotype control mAb were sacrificed early for non-treatment related welfare reasons (cracked tail and 20% drop in bodyweight, respectively) and they were not included in the analyses.

#### Plasma samples

Plasma samples were collected as previously described in the single dose study.

#### Processing of the brain tissue

Whole brain homogenates were prepared as described in the single-dose study with the exception that TBS buffer was used instead of RIPA buffer to prevent interference in assays involving Tau RD P301S FRET biosensor cells.

For seed-competent tau, an aliquot of brain homogenate was sonicated (5 × 4 s cycles (60% amplitude, 0.5 mm probe (VC50T Vibra-Cell Sonicator))) prior to processing.

For the quantification of insoluble phosphorylated tau, a pellet obtained by differential centrifugation of brain homogenate in TBS was used. The homogenate was centrifuged at 10,000 × g for 10 min and the supernatant centrifuged at 200,000 × g for 30 min. The resultant pellet was further washed with TBS and centrifuged as before. The final pellet was sonicated in TBS for 5 × 4 s cycles (60% amplitude, 0.5 mm probe (VC50T Vibra-Cell Sonicator)) and used for the measurement of insoluble phosphorylated tau as described below.

#### Measurement of aggregated tau in brain homogenate

A black Nunc MaxiSorp flat-bottom 96-well plate (Fisher, #10394751) was coated with 2.5 µg/mL of CB7, an antibody that recognises an N-terminal epitope of tau (tau13-25; [19]) and blocked with StartingBlock™ Blocking Buffer (Thermo, #37538) for 1 h at 37 °C. Single murine brain homogenate samples were added to the plate at a concentration of 100 µg/mL total protein in PBS [19]. Sarkosyl-insoluble samples containing tau filaments were isolated from AD patient frontal cortex as previously described [19, 27] and used as a calibrator to generate a standard curve for this assay using protein concentrations ranging from 600–0.82 ng/mL (determined via BCA assay). The calibrator and samples were each diluted in 20% SuperBlock™ Blocking Buffer (Thermo, #37515) in PBS and incubated for 1 h at 37 °C. This solution was used as diluent for subsequent steps. An ELISA protocol was followed as above with the addition of a biotinylated detector antibody (CB7, 200 ng/mL), followed by streptavidin poly-HRP (1:20,000, Thermo, #21140) before addition of SuperSignal™ ELISA Femto Substrate Solution (Thermo Scientific, #37074) and the reading of luminescence on a Clariostar Plus microplate reader (BMG Labtech) to quantify aggregated tau from mouse brain homogenates.

#### Measurement of insoluble phosphorylated tau in brain homogenate

A sonicated high-speed pellet (prepared as above) was used to determine insoluble phosphorylated tau. Tau phosphorylated at Ser-396 (p396) was determined using Invitrogen™ KHB7031 Tau (Phospho) [pS396] Human ELISA Kit following the manufacturer’s instructions. Samples were quantified using a 4-parameter-fit standard curve with calibrators run in duplicate and single samples (1 in 5 dilution).

Levels of tau phosphorylated at Ser-202 and Thr-205 (p202/205) was determined by AT8 (Thermo #MN1020) antibody using a standard western blot (WB) method [28]. Brain homogenates (10 µg) from each mouse were separated by SDS-PAGE (CriterionTM, Biorad, #165600) using 1 × Tris–glycine-SDS running buffer. Brain homogenate samples were prepared in Laemmli buffer (Thermo, #J61337) under reducing conditions and heated at 95 °C for 10 min.

Proteins were subsequently transferred from SDS-PAGE gels onto a 0.2 µm nitrocellulose membrane (Trans-Blot® Turbo™ RTA Midi Nitrocellulose Transfer Kit, Biorad, #1704271) as per manufacturer’s specifications. The membranes were blocked in TBS containing 0.1% Tween® 20 (TBS-T) with 5% (w/v) bovine serum albumin (BSA, Merck, #A3059) for 1 h at room temperature prior to overnight incubation at 4 °C with AT8 (Thermo, #MN1020) with AC-74 (anti-beta-actin antibody, Merck #A2228, 1:5000) included as a loading control. The membranes were washed three times with TBS-T for 5 min per wash and then incubated for 1 h with sheep anti-mouse IgG-HRP (Merck, #A6782, 1:2,000) in TBS-T with 5% (w/v) BSA. The membranes were washed as before and developed with Clarity Western ECL Substrate (Bio-Rad, #1705061) using a BioSpectrum Gel Imaging System (UVP). Results were analysed using ImageJ-Fiji (NIH, Version 1.53); densitometric analysis was performed on p-tau and beta-actin bands.

#### Seed-competent tau assessment in brain homogenate

Seed-competent tau was assessed via addition of sonicated brain homogenate to Tau RD P301S FRET biosensor cells [29]. Tau RD P301S FRET cells were incubated, transfected with brain homogenate and prepared for quantitative assessment of tau seeding via flow cytometry, as previously reported [19].

#### Measurement of core tau in plasma

Two separate Simoa homebrew core-tau assays were developed as part of this study and used for mouse plasma analysis. These three-step Simoa assays are termed core-proline tau and core-core tau, respectively. A summary of Simoa antibody beads, detector antibodies and mouse plasma dilutions for each tau analysis in mouse plasma is provided in Table 2.

**Table 2:** Details of Simoa assays for analysis of tau in mouse plasma

Simoa assay	Antibody on capture beads (tau epitope)	Biotinylated detector antibody (tau epitope)	Mouse plasma dilution
Core-Proline	S1G2 (tau367-379)	BT2 (200 ng/mL) (tau194-198)	1 in 50
Core-Core	CA4 (tau355-367)	S1G2 (10 ng/mL) (tau367-379)	1 in 8

Tau epitope numbering corresponds to that of the human 2N4R tau isoform

All calibrators, in triplicate, and mouse samples, in duplicate, (final volume 100 µL) were added to a Simoa SR-X sample plate (Quanterix #103022) in Tau 2.0 diluent (Quanterix #103847). Calibrators consisted of full-length human 2N4R tau to generate a standard curve ranging from 540–0.74 pg/mL for the core-proline assay and 10,000–41.2 pg/mL for the core-core assay. Subsequently, 25 µL of 5 × 10^6^ capture beads were added to each well, consisting of 25% antibody-coated beads and 75% blocked helper beads for both assays (Quanterix #103208).

The three-step Simoa protocol was followed according to the manufacturer’s instructions. Plasma tau concentrations were quantified using a 4-parameter-fit standard curve.

#### Measurement of neurofilament light chain (NfL) and phosphorylated tau (pTau217) in plasma

Assessment of NfL and pTau217 was performed using Simoa NF-Light v2 Advantage Reagent Kit (#104364, Quanterix) and ALZpath Simoa pTau217 Assay kit (#104570, Quanterix), respectively, following manufacturer’s instructions and using a Simoa SR-X reader. Samples were diluted 20-fold and performed as single assays.

#### Assessment of therapeutic potential of S1D12 across various tauopathies

Frozen brain tissue (Table 3) from the Cambridge Brain Bank (CBB, Cambridge University Hospitals NHS Foundation Trust of Addenbrooke’s Hospital Hills Road, Cambridge) was derived from histopathologically confirmed cases of tauopathies. Samples were taken from Brodmann area 20 (temporal region), a region that contains tau pathology across all these diseases.

**Table 3:** Details of brain samples representing various primary tauopathies

BB Number	Age (yr)	Sex	PMI (h)	Diagnosis
BB16.0028	91	F	34	AD
BB16.0039	78	M	44	AD
BB20.0010	80	M	31	AD
BB321	72	M	40	AD
PT209	83	M	59	bvFTD/Picks
BB19.0021	73	M	37	bvFTD/Picks
BB21.0017	67	M	67	bvFTD/Picks
BB23.0024	74	F	102	bvFTD/Picks
BB16.0004	82	F	37	PSP
BB19.0004	88	M	88	PSP
BB19.0048	85	F	32	PSP
BB22.0027	78	F	121	PSP
BB18.0019	87	F	47	CBD
BB17.0006	80	M	43	CBD
BB17.0023	86	F	62	CBD
BB19.0067	75	M	74	CBD
BB19.0046	68	F	85	AGD

*AD* Alzheimers disease, *bvFTD* behavioural variant frontotemporal dementia, *PSP* progressive supranuclear palsy, *CBD* corticobasal degeneration, *AGD* Argyrophilic grain disease, *F* female, *M* male, *PMI* postmortem interval

Preparation of human brain homogenates and assessment of anti-tau seeding activity of S1D12 against tauopathies was performed via protein A-based immunodepletion with the resulting supernatant being transfected into Tau RD P301S FRET cells [29] with subsequent flow cytometry to assess levels of tau seeding according to methods described [19].

### Statistical analysis

All statistical analyses were carried out using Prism version 10.4.2. (GraphPad Software Inc., San Diego, USA). Results are expressed as individual values and group mean ± SD. Normality testing was performed by Shapiro–Wilk test and QQ plot to help determine suitability of parametric (ANOVA with Tukey multiple comparison tests, or Student’s t-test for two groups) or non-parametric statistics (Kruskal–Wallis test for overall comparisons followed by Dunn’s multiple comparisons test or Mann Whitney test for comparison between two groups). Our statistical approach started with an overall comparison of all treatment/age conditions, which was followed by a subsequent in-depth profiling of the two age cohorts separately (*young*: Baseline-2 month, Negative-5 month and S1D12-5 month; *old*: Baseline-4.5 month, Negative-7.5 month and S1D12-7.5 month). Linear regression and correlation (Spearman nonparametric correlation) analyses were carried out to examine the relationship between biochemical endpoints. Data were converted into heat ma1455 nM. This is comparable with otherps expressing all correlative analyses and two-parameter correlations were expressed as point-diagrams. Prism was used to fit non-linear 4-parameter logistic regression models for NfL and measures of brain tau pathology. For all comparisons, a 95% confidence level (*p* < 0.05) was set for the differences to be considered as significant. Only significant terms are mentioned for clarity.

## Results

### Single dose study – S1D12 demonstrates a long half-life and brain penetration

By utilising the ultra-high affinity of S1D12 to dGAE, a sensitive ELISA-based method was developed to quantify the amount of free S1D12 in plasma, brain and peripheral tissues.

**Fig. 2:**
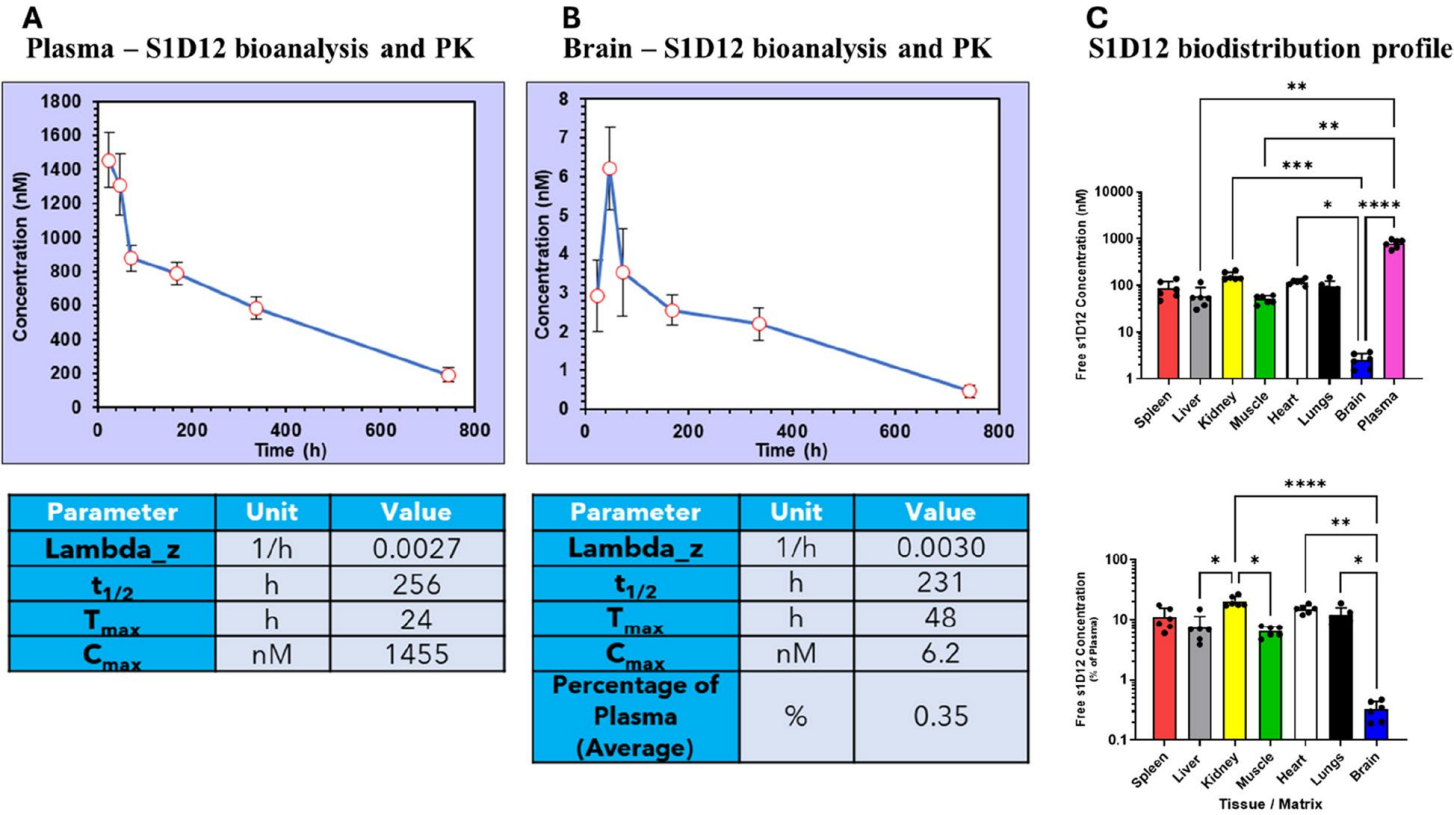
Pharmacokinetic and tissue distribution profile of S1D12 from a single-dose study. Pharmacokinetic parameters were calculated using PK Solver using non-compartmental analysis following a log-linear trapezoidal method. Individual mouse concentrations were determined from 3 individual ELISA experiments in which each sample was tested in duplicate (*n* = 6 mice per timepoint). **A** Single i.p. dose pharmacokinetic profile of S1D12 (linear scale) in plasma and calculated pharmacokinetic parameters. **B** Single i.p. dose pharmacokinetic profile of S1D12 (linear scale) in brain and calculated pharmacokinetic parameters. Lambda_z = terminal elimination rate constant, t1/2 = terminal half-life, Tmax = time point of maximum observed concentration, Cmax = maximum observed concentration. **C** S1D12 biodistribution in plasma, brain and peripheral tissues (top panel, free S1D12 concentration (nM); bottom panel, free S1D12 concentration (% of plasma)). All values expressed as mean + SD. Data were analysed using Kruskal Wallis non-parametric statistics followed by Dunn’s multiple comparison test (*, *p* < 0.05; **, *p* < 0.01; ***, *p* < 0.001; ****, *p* < 0.0001)

The pharmacokinetics of S1D12 in plasma (Fig. 2A) revealed absorption from the intraperitoneal space with T_max_ being the first time point tested (24 h), resulting in a C_max_ of 1455 nM. This is comparable with other tau antibody administration by i.p., where a T_max_ of 6 h was reported, albeit in rats [30]. At subsequent time points, S1D12 concentration decreased in plasma. Terminal half-life (t_1/2_) calculations revealed a relatively long period of 256 h (10.7 days), that was broadly comparable to other tau antibodies such as armanezumab (N-terminal; 9.7 days), administered i.v. in mice [31].

The pharmacokinetic analysis showed detectable levels of S1D12 in the brain (Fig. 2B). The T_max_ and C_max_ were 48 h and 6.2 nM, respectively and the average concentration in the brain compared to plasma across the time course was 0.35%. This value was slightly higher than the levels reported for other tau antibodies, with the maximum being 0.31% (DMR7, [32]). The t_1/2_ for S1D12 in brain was 231 h (9.6 days), which was broadly comparable to the t_1/2_ in blood.

The biodistribution of S1D12 in peripheral tissue was quantified in all tissues measured including spleen (85.9 nM, 10.8%), kidney (160.4 nM, 20.3%), liver (58.2 nM, 7.4%), muscle (50.6 nM, 6.4%), heart (119.1 nM, 15.1%) and lungs (94.4 nM, 12.0%) (Fig. 2C Top). Values differed significantly between groups (X^2^(7,48) = 41; *p* < 0.0001). Of particular note here is the low uptake of free s1D12 into brain while levels in other tissues were all higher (kidney ~ 20%, heart ~ 15%; Fig. 2C Bottom; X^2^(6,42) = 33.3; *p* < 0.0001). These quantities and proportions relative to plasma are broadly comparable to the established literature for biodistribution of a monoclonal antibody [33, 34].

### Plasma and brain concentrations of S1D12 are similar in both young and old mice

**Fig. 3:**
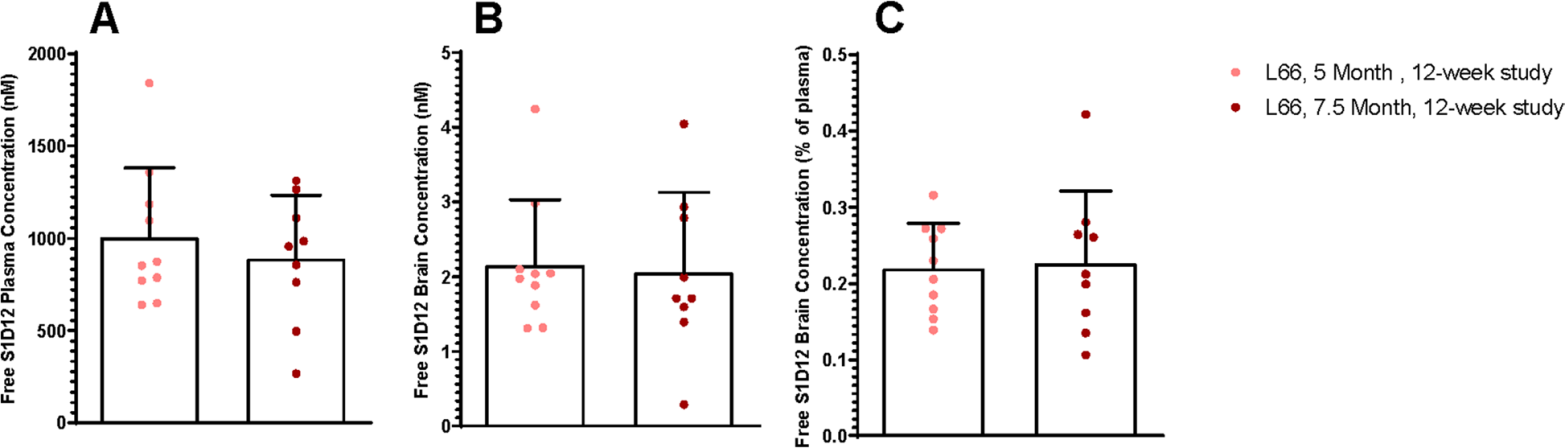
S1D12 plasma and brain concentrations from 12-week study. S1D12 **A** plasma and **B** brain concentrations in L66 mice following 8 doses. **C** S1D12 brain concentration relative to plasma concentration in L66 mice. Values expressed as mean + SD. Data were analysed with Two-tailed t test (**A** and **C**) and Mann Whitney test (**B**) (no significant difference were detected; *p* > 0.05)

To determine exposure of S1D12 in L66 mice in a 12-week proof-of-concept study, plasma and brain samples were analysed by ELISA to determine free mAb concentration in these matrices one week after the final dose (Fig. 3). L66 mice at 5 months and 7.5 months of age had similar S1D12 concentrations in both plasma (1005 ± 375 nM and 890 ± 342 nM, respectively) and brain (2.15 ± 0. 87 nM and 2.05 ± 1.08 nM, respectively). One plasma sample in the 7.5-month −12-week study group was identified as an outlier (Z:2.29; *p* < 0.05) and all data for this sample were excluded from analysis.

When comparing S1D12 concentrations in the brain of L66 mice (0.22 ± 0.06% and 0.23 ± 0.10% for 5-month and 7.5-month cohorts, respectively, expressed as a percentage of plasma levels), there was no difference. This confirms a robust uptake of S1D12 over 12 weeks independent of the pre-existing level of tau or the age of animals. Whilst low, the levels for mAb delivered to the L66 brains are still within the range reported elsewhere (0.1–0.3%). S1D12 was not detected in animals at baseline or in animals receiving negative control treatment (data not shown).

### S1D12 prevents accumulation of aggregated tau and seed-competent tau in L66 mice (12-week study)

**Fig. 4:**
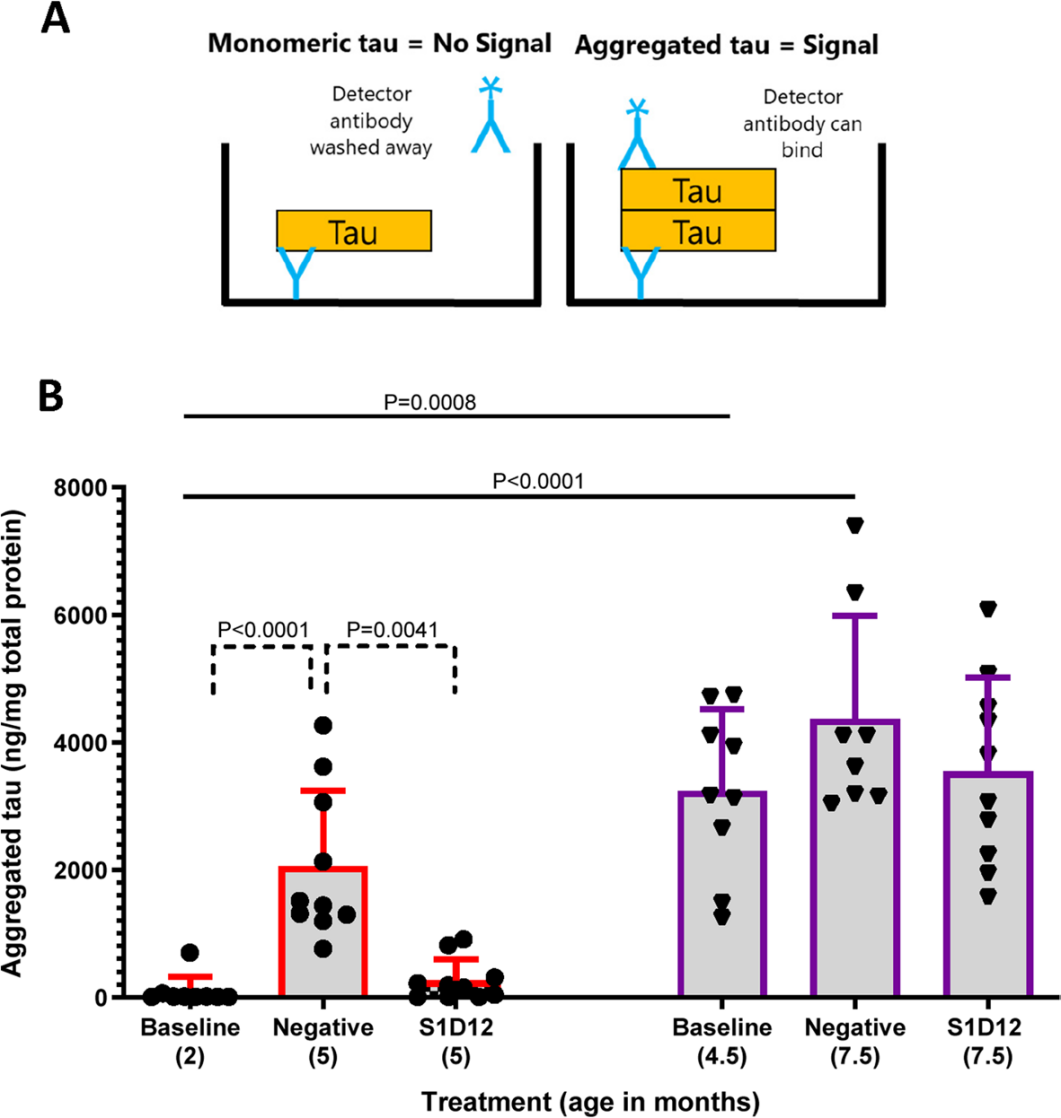
Effect of S1D12 treatment on aggregated tau in L66 mice brain. **A** Schematic representation of mono-antibody ELISA used to measure aggregated tau (inclusive of all aggregated tau species, from small oligomeric to large filamentous forms). mAb CB7, that recognises an N terminal epitope of tau, was used as both capture and detector antibody in this assay. Since CB7 can only bind one epitope per tau molecule, a binding reaction is only detected in the presence of aggregated tau. **B** Aggregated tau levels in study groups. Negative: isotype control mAb B11. Values expressed as mean + SD; age denotes age at sacrifice. Kruskal–Wallis test with Dunn’s multiple comparison test was performed among all groups (solid line) and between young and old cohorts independently (dashed line)

To monitor aggregated tau in L66 brain homogenates, a mono-antibody ELISA (adapted from [35]) was developed. This utilises the principle that by using the same capture and detector antibodies in a sandwich ELISA format, only tau that is aggregated will have multiple epitopes and be quantified. This measurement will be inclusive of all aggregated tau species, from small oligomeric to large filamentous forms (Fig. 4A).

**Fig. 5:**
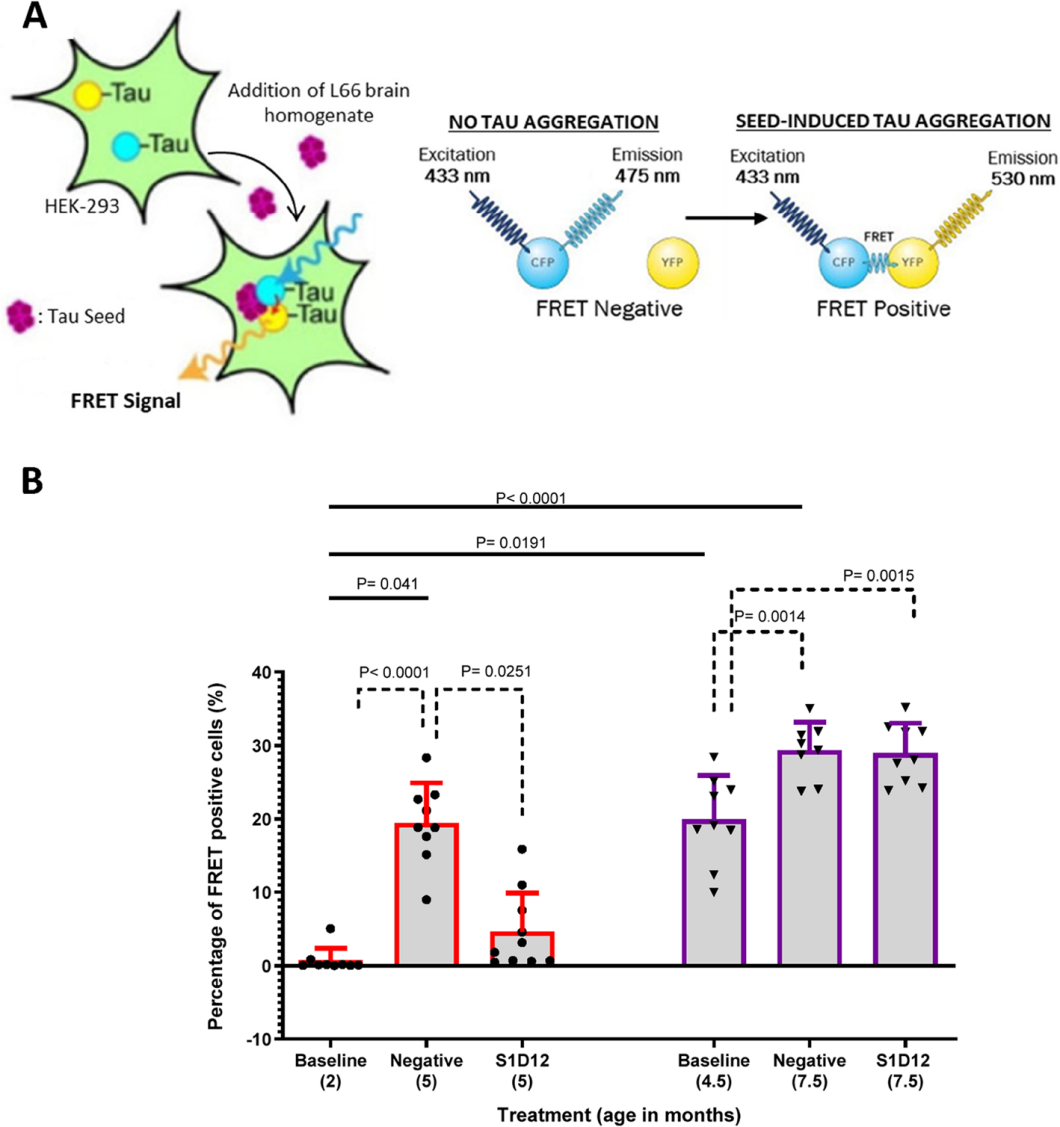
Effect of S1D12 treatment on seed competent tau in L66 mouse brain. **A** The presence of tau seeds results in propagation and initiation of aggregation of the two tau constructs in Tau RD P301S FRET biosensor cells. CFP can be excited with a laser causing YFP emission due to the energy transfer of these being in close proximity thus allowing cells to be classed as FRET positive or negative (adapted from [37]). **B** Seed competent tau levels in L66 study groups. Negative: isotype control mAb B11. Values expressed as mean + SD; age denotes age at sacrifice. Kruskal–Wallis test with Dunn’s multiple comparison test was performed among all groups (solid line). Dashed lines indicate comparison between young and old cohorts analysed independently (young: Kruskal–Wallis test with Dunn’s multiple comparison test; old: ANOVA and Tukey’s multiple comparison test)

Furthermore, cell to cell propagation of tau resulting in the spread of tau pathology is a key process in tauopathies [29, 36] and the ability of antibodies to reduce ‘seed competent’ tau is pivotal for tau immunotherapies to be effective. To monitor seed competency of tau in L66 mice, brain homogenates were added to HEK Tau RD P301S FRET biosensor cells and the percentage of FRET-positive cells quantified using flow cytometry (adapted from [29]). In brief, Tau RD P301S FRET biosensor cells carry constructs encoding tau RD P301S-CFP and tau RD P301S-YFP [29]. When tau seeds (in this case, endogenously derived aggregation competent tau) are introduced into these cells, aggregation of the two tau constructs is initiated and excitation of tau-CFP cause tau-YFP emission due to their close proximity (Fig. 5A).

In the younger cohort of mice, aggregated tau levels increased 22-fold, and seed-competent tau increased 29-fold over the 12-week treatment period. Specifically, aggregated tau levels increased from 93.4 ± 228.6 ng/mg of total protein at baseline in 2-month-old mice to 2060 ± 1182 ng/mg in 5-month-old negative control mice (Fig. 4B). Similarly, seed-competent tau, measured by % FRET-positive cells, increased from 0.73 ± 01.64% at baseline in 2-month-old mice to 19.4 ± 5.4% in 5-month-old negative control mice (Fig. 5B). Overall Kruskal–Wallis tests for non-parametric data returned a main effect of group for both aggregated and for seed competent tau (X^2^ values > 41; *p* values < 0.0001). Multiple comparisons reported specific age-related increases in the non-S1D12 cohorts (Figs. 4B and 5B; solid lines). A post-hoc planned comparison was performed for each age cohort. There was a significant main effect of treatment for both tau species (X^2^ values > 19, *p* values < 0.0001) with a strong S1D12 effect reducing tau to levels near the detection threshold (Figs. 4B and 5B, dotted lines).

In the older cohort of mice, aggregated tau increased 1.3-fold, and seed-competent tau increased 1.5-fold over the treatment period. Specifically, aggregated tau levels increased from 3239 ± 1277 ng/mg of total protein at baseline in 4.5-month-old mice to 4366 ± 1621 ng/mg in 7.5-month-old negative control mice (Fig. 4B). Similarly, seed-competent tau, measured by % FRET-positive cells, increased from 19.9 ± 5.97% at baseline in 4.5-month-old mice to 29.3 ± 3.85% in 7.5-month-old negative control mice (Fig. 5B). This increase in seed-competent tau was significant (see Fig. 5B, dotted lines; *F*(3,26) = 11; *p* = 0.0004). Due to biological variance and the modest changes observed in this older cohort, detecting inhibitory treatment effects was challenging and levels remained high for aggregated (3544 ± 1472 ng/mg) and seed-competent tau (28.95 ± 4.1%) with no significant S1D12 effects observed.

The primary objective and inclusion of the older cohort of mice was not to only look for inhibition of tau aggregation but also to determine whether S1D12 can remove already established tau pathology below baseline. This capability was not observed given the minimal increase in aggregated and seed competent tau from baseline in S1D12-treated animals.

### S1D12 prevents accumulation of insoluble phosphorylated tau in L66 mice brain (12-week study)

Although phosphorylation mediates the physiological function of tau by regulating its binding to microtubules [30], abnormal hyperphosphorylation of specific residues within PHFs and insoluble tau is characteristic of AD and tauopathies. Two well established examples being p396 [38] and p202/205 [39] which are archetypal of disease-relevant tau.

**Fig. 6:**
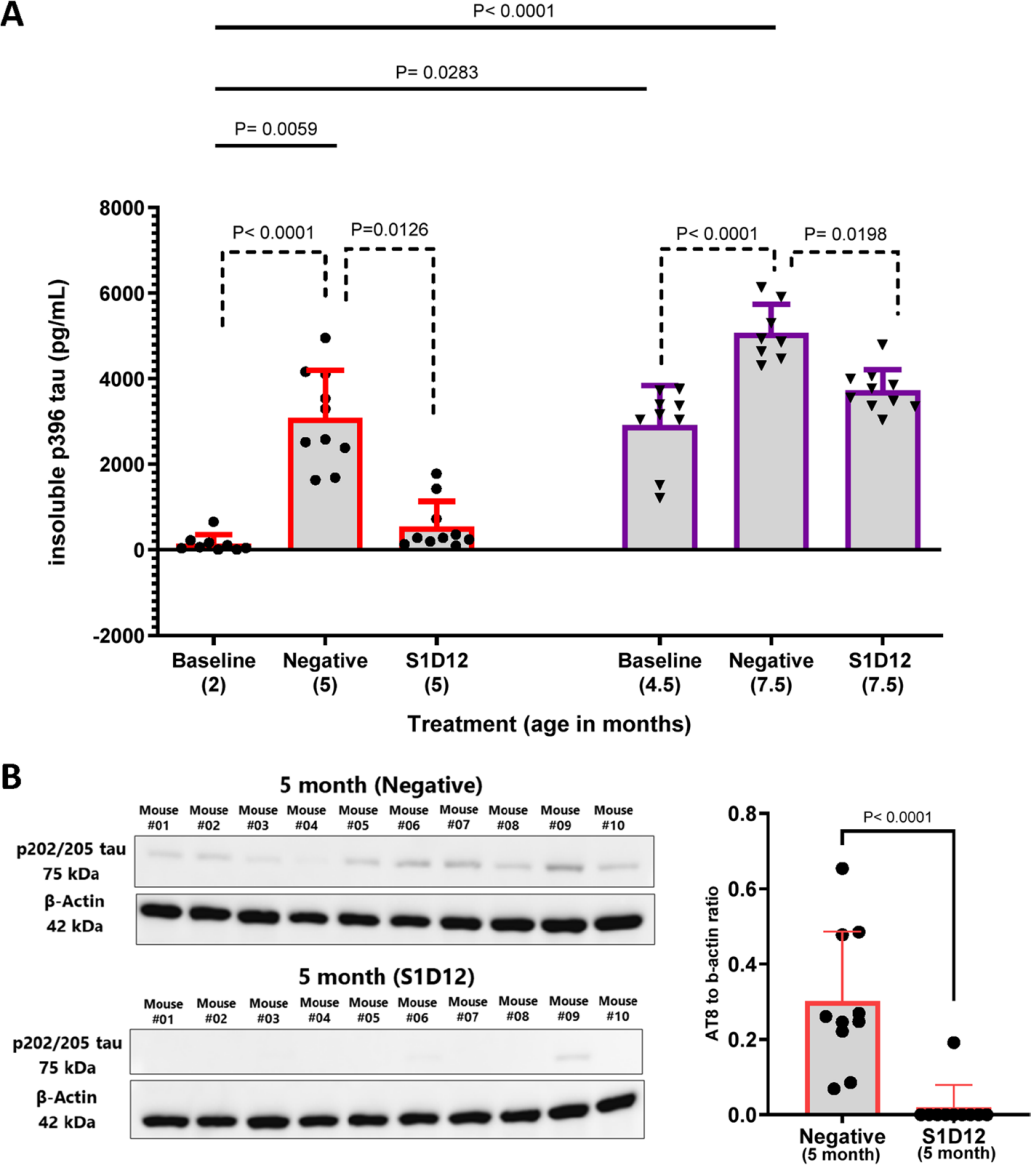
Effect of S1D12 treatment on insoluble phosphorylated tau in L66 mice brain in12-week study. **A** Insoluble p396 tau levels in study groups determined using commercial ELISA kit (Invitrogen™ KHB7031 Tau (Phospho) [pS396] Human ELISA Kit). Values are expressed as mean + SD; age denotes age at sacrifice. Kruskal–Wallis test with Dunn’s multiple comparison test was performed among all groups (solid line) and between young and old cohorts independently (dashed line). **B** Westen blot images (left) of 5-month-old L66 mice brain homogenates, where p202/205 was detected using AT8 antibody (1:500). ImageJ software was used for densitometric analysis (right). Age denotes age at sacrifice. Mann Whitney test was performed between treatments. Values expressed as mean + SD. In the older cohort of mice, no increase of p202/205 was evident on comparison to baseline nor was any treatment effect evident (data not shown)

The varying levels of phosphorylated tau detected in the different age and treatment groups yielded a main effect of group in the overall Kruskal–Wallis test (X^2^(6,56) = 45, *p* < 0.0001). Similar to aggregated and seed-competent tau levels, p396 tau increased by age (Fig. 6A, solid lines). In the younger cohort of mice, there was a 29-fold increase in insoluble p396 tau across the treatment period when comparing 2-month-old baseline mice (138.6 ± 203.9 pg/mL) with 5-month-old negative control mice (3081 ± 1110 pg/mL) and this was reduced to baseline levels when S1D12 was administered (544.4 ± 586.1 pg/mL, Fig. 6A, dotted lines: X^2^(3,29) = 21, *p* < 0.0001).

In the older cohort of mice, there was a 1.7-fold increase in insoluble p396 tau across the treatment period when comparing the 4.5-month-old baseline mice (2910 ± 920 pg/mL) with 7.5-month negative control mice (5067 ± 666 pg/mL, Fig. 6A). This age-related increase was prevented by the presence of S1D12 (3723 ± 491 pg/mL, Fig. 6 A, dotted lines; X^2^(3,27) = 17.6, *p* = 0.0002). However, there was once again no evidence of removal of already established aggregated tau given the modest increase from baseline in S1D12 treated mice (812 ± 155 pg/mL).

To further explore these findings, western blotting with mAb AT8 was performed on these brain homogenate samples. When comparing 5-month-old S1D12 treated mice with the 5-month-old negative control, there was a 97% difference (Mann–Whitney, *p* < 0.0001) in insoluble p202/205 tau between these groups (Figs. 6B and S3). However, unlike insoluble p396 tau, there were no statistical differences between the 4.5-month-old baseline, 7.5-month-old negative control and 7.5-month-old S1D12-treated mice (data not shown). This strongly suggests that S1D12 is unable to affect established pathology under these circumstances and that this assay is unsuitable for measuring the progression of insoluble p202/205 tau in L66 mice of this age. Furthermore, this also suggests that the temporal change in insoluble p396 and p202/205 tau does differ and that this is more dynamic using p396 within the older age group of mice.

### S1D12 inhibits plasma pTau217 in L66 mice (12-week study)

Blood-based biomarkers for AD have advanced greatly in recent years with plasma pTau217 proving to be the best singular tau biomarker of AD biology. Studies have shown plasma pTau217, increases in preclinical stages of AD, progresses longitudinally with disease severity and provides better correlation with tau pathology when compared to other pTau assays [40–42]. However, plasma pTau217 remains unexplored in murine models of tauopathy.

Neurodegeneration is a key downstream consequence of tau pathology and is fundamental to AD and other primary tauopathies [43, 44]. Among the fluid-based biomarkers representing neurodegeneration, NfL is one of the most promising (and utilised) markers of axonal neurodegeneration [45, 46]. In most neurodegenerative diseases, including AD and other tauopathies, higher levels of plasma NfL represents both faster disease progression and increased rates of brain atrophy [47, 48]. Therefore, plasma NfL may serve as a surrogate biomarker for neurodegeneration even in the prodromal stages of AD ([49], Fig. 8A).

**Fig. 7:**
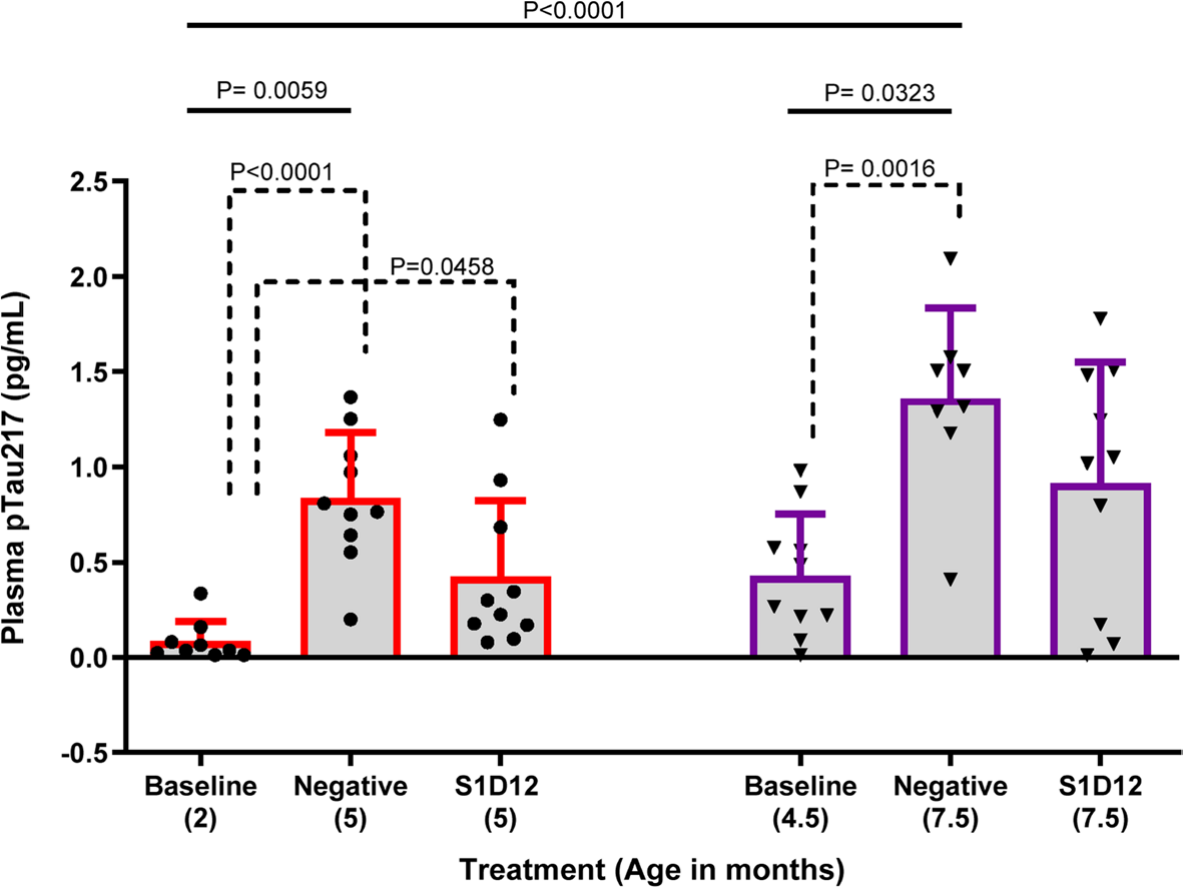
Effect of S1D12 on plasma pTau217 in L66 mice. Plasma pTau217 levels in groups determined by commercially available ALZpath Simoa pTau217 Assay Kit as per manufacturer’s instructions. Kruskal–Wallis test with Dunn’s multiple comparison test was performed among all groups (solid line). Dashed lines indicate comparison between young and old cohorts analysed independently (young: Kruskal–Wallis test with Dunn’s multiple comparison test; old: ANOVA and Tukey’s multiple comparison test). Values are expressed as mean + SD; age denotes age at sacrifice

**Fig. 8:**
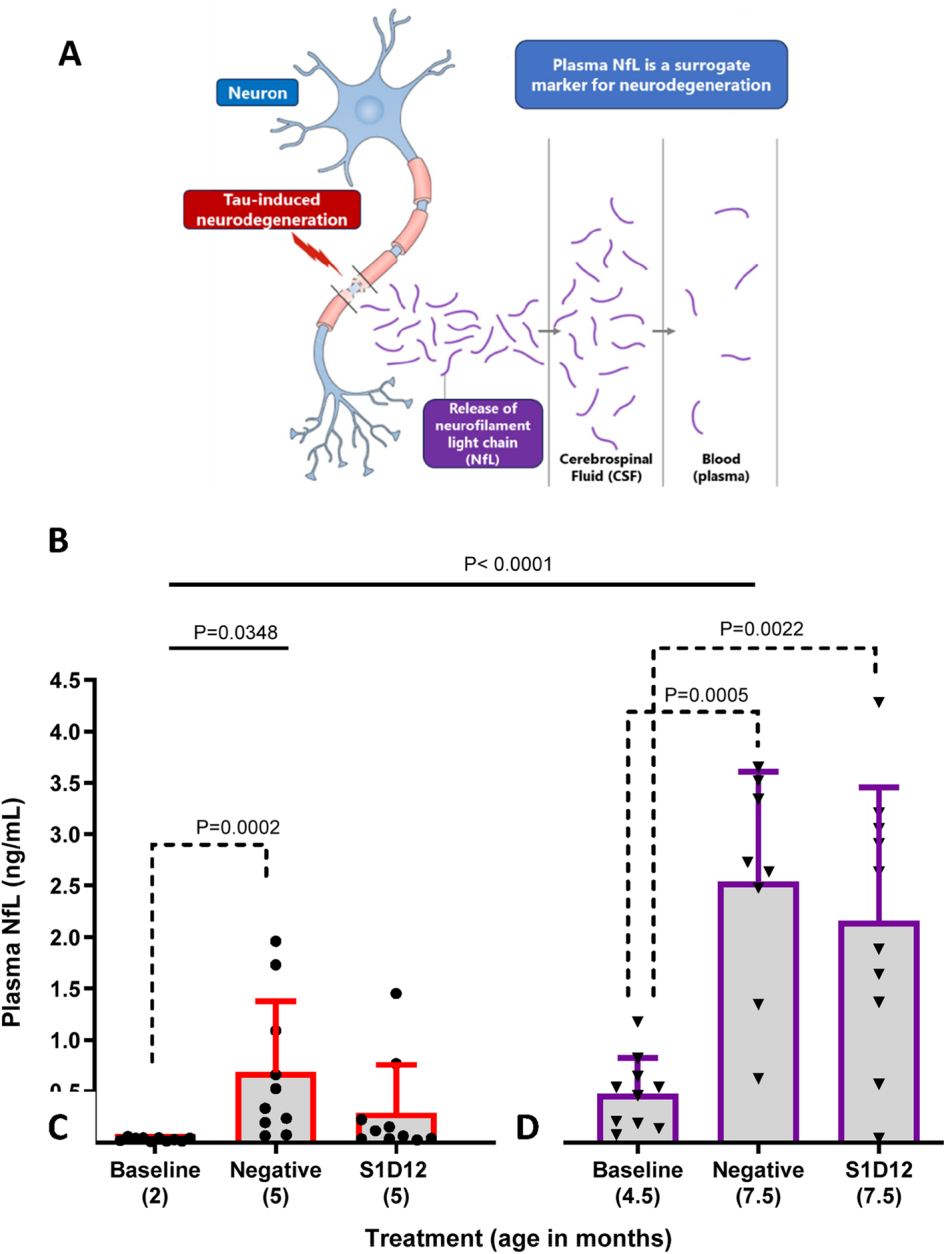
Effect of S1D12 on plasma NfL. **A** Schematic depicting release of NfL from degenerating neurons and its measurement in plasma is a surrogate marker for this process (Adapted from [47]). **B** Plasma NfL levels in groups was determined by commercially available Simoa NF-Light v2 Advantage Reagent Kit as per manufacturer’s instructions. Kruskal–Wallis test with Dunn’s multiple comparison test was performed among all groups (solid line). Dashed lines indicate comparison between young and old cohorts analysed independently (young: Kruskal–Wallis test with Dunn’s multiple comparison test; old: ANOVA and Tukey’s multiple comparison test). Values expressed as mean + SD; age denotes age at sacrifice

In this study, there was considerable variability between age and treatment group for pTau217 (X^2^(6,57) = 28.7, *p* < 0.0001) and NfL (X^2^(6,58) = 37.7, *p* < 0.0001). Both 5 and 7.5-month-old negative controls presented with heightened plasma pTau217 and plasma NfL (Figs. 7 and 8, solid lines). The younger cohort of mice experienced a 10-fold increase in plasma pTau217 and a 21-fold increase in plasma NfL over the 12-week treatment period. Specifically, pTau217 increased from 0.084 ± 0.105 pg/mL at baseline in 2-month-old mice to 0.84 ± 0.34 pg/mL in 5-month-old negative control mice (Fig. 7A, X^2^(2,27) = 18.8, *p* < 0.0001). Although a clear reduction to 0.33 ± 0.29 pg/mL was noted for the S1D12 treatment, this did not show significance due to some abnormally high and low values in the two cohorts (Fig. 7A, dotted lines). Similarly, plasma NfL (young cohort: X^2^(3,30) = 15.9, *p* = 0.0004) increased from 0.033 ± 0.017 ng/mL at baseline in 2-month-old mice to 0.69 ± 0.68 ng/mL in 5-month-old negative control mice and remained at baseline in the 5-month-old S1D12 cohort (0.29 ± 0.46 ng/mL) (Fig. 8B). Although the data only showed a trend towards a reduction (*p* = 0.15), the blockade of NfL increase is further confirmed by the lack of difference between 2-month-old controls and the 5-month-old S1D12 mice.

In the older cohort of mice, pTau217 increased 3.2-fold and plasma NfL increased 5.3-fold across the treatment period. Specifically, pTau217 increased from 0.43 ± 0.32 pg/mL at baseline in 4.5-month-old mice to 1.36 ± 0.47 pg/mL in 7.5-month-old negative control mice (Fig. 7A) and was marginally lower in the 7.5-month-old S1D12 samples (0.91 ± 0.63 pg/ml). Despite a significant group difference in the ANOVA (*F*(2,25) = 7.9, *p* = 0.002), the lowering in the S1D12 group was not significant (t = 1.6, df = 16, *p* > 0.1). Similarly, plasma NfL increased from 0.48 ± 0.35 ng/mL at baseline in 4.5-month-old mice to 2.54 ± 1.07 ng/mL in 7.5-month-old negative control mice, and this was not lowered in 7.5-month-old S1D12 mice (2.2 ± 1.3 ng/mL; Fig. 8B). Although an overall main effect of group was returned (*F*(2,25) = 11.7, *p* = 0.0003) there was again no difference between the two treatment groups (t < 1).

Consistent with other biomarkers in this older cohort, biological variance made it challenging to observe a treatment effect and there was no evidence of removal of pre-existing tau pathology or reduction in plasma NfL below baseline, given the increase from baseline in S1D12-treated mice.

### Effect of S1D12 on plasma core-tau fragments in L66 mice (12-week study)

**Fig. 9:**
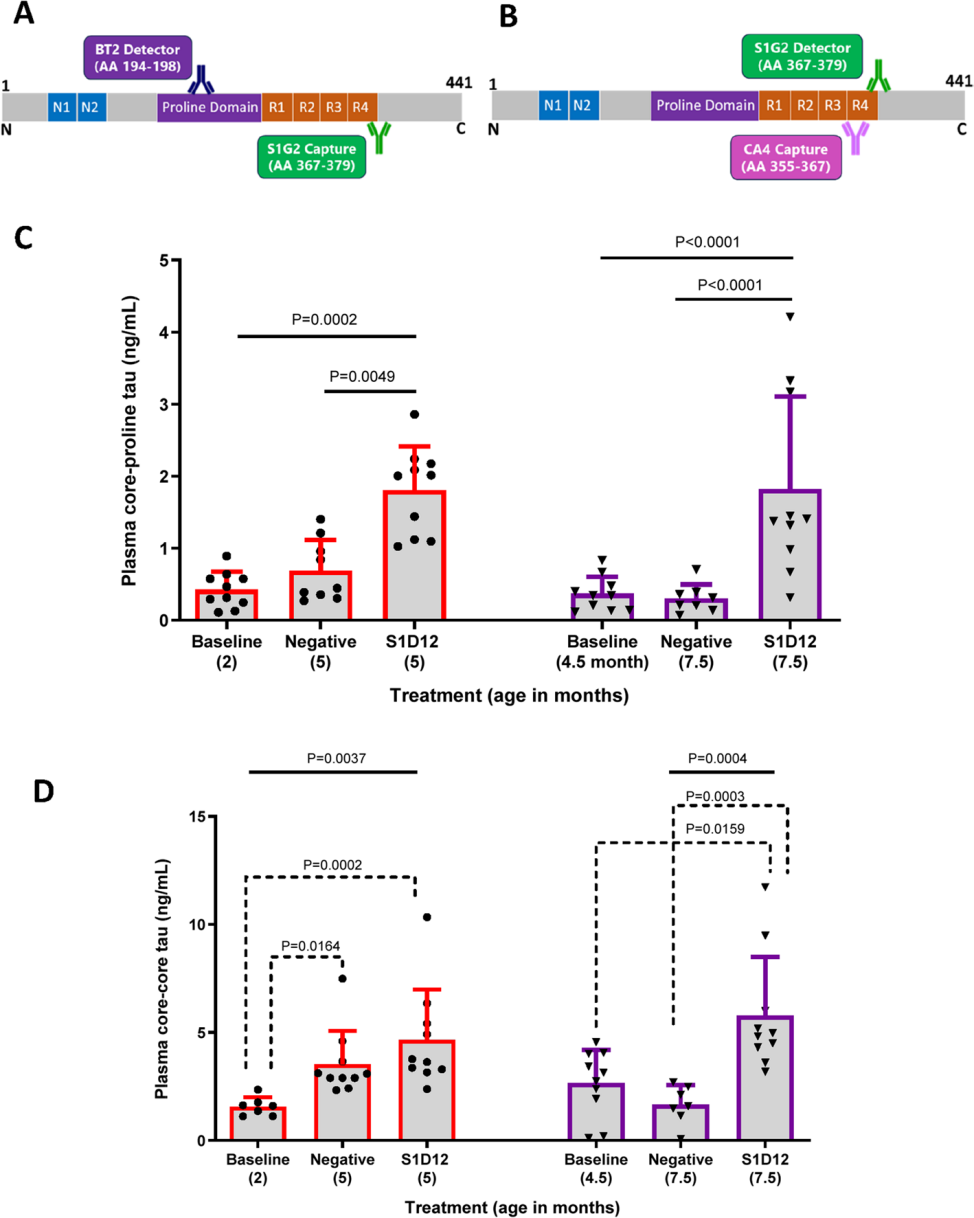
Effect of S1D12 on plasma core-tau. Schematic representation of antibody pairs and their tau epitopes used in Simoa assays for **A**, **C** core-proline and **B**, **D** core-core tau with the respective data. AA (amino acid) followed by numbers refers to binding epitopes of mAbs (based on numbering of the 2N4R tau isoform). **C**) Two-way ANOVA and Tukey’s multiple comparison test was performed among all groups (solid line). **D**) Kruskal–Wallis test with Dunn’s multiple comparison test was performed among all groups (solid line) and between young and old cohorts independently (dashed line). Values expressed as mean + SD; age denotes age at sacrifice

To further understand the ability of S1D12 to engage with tau in vivo, two core-tau assays were developed using the Simoa technology platform to detect tau fragments in plasma. The core-proline tau assay (Fig. 9A) utilises S1G2 as capture antibody (to a ‘core’ epitope, tau367-379) and BT2 (to a ‘proline domain’ epitope, tau194-198) for detection. The core-core tau assay (Fig. 9B) uses CA4 as capture antibody (to a ‘core’ epitope, tau 355–367) and S1G2 (‘core’ epitope, tau 367–379) for detection.

When making comparisons to age-matched mice receiving negative control treatment, core-proline tau (Fig. 9C) was 2.6-fold higher in 5-month-old mice receiving S1D12 (0.686 ± 0.42 vs. 1.806 ± 0.61 ng/mL, respectively) and 6.1-fold higher in 7.5-month-old mice (0.299 ± 0.197 vs. 1.824 ± 0.128 ng/mL, respectively). A 2-way ANOVA with age and treatment as independent factors confirmed that there was a main effect of treatment, but no effect of age or interaction. This confirmed the impression that, independent of age, levels of plasma core-proline tau are low in untreated cohorts and that S1D12 treatment led to a release of tau into plasma (Fig. 9C for detailed statistics).

Similarly, when compared to age-matched mice receiving negative control treatment, core-core tau (Fig. 9D) was 1.3-fold higher in 5-month-old mice receiving S1D12 (3.537 ± 1.53 vs. 4.660 ± 2.32 ng/mL, respectively) and 3.5-fold higher in 7.5-month-old mice (1.662 ± 0.89 vs. 5.777 ± 2.71 ng/mL, respectively). This non-parametric data set was analysed by Kruskal–Wallis (overall: X^2^(6,54) = 32.3, *p* < 0.0001) and the planned analyses of the age groups also showed significant differences between the groups (X^2^ values > 16; *p* values < 0.0004). While baseline levels were more variable than for core-proline tau, the core-core tau values nevertheless were significantly highest following S1D12 treatment in both young and old cohorts.

There was no evidence of temporal progression for either of these biomarkers and thus these assays only serve as markers of target engagement and the data for baseline groups are shown for reference only.

### Correlation analyses between endpoints monitoring tau pathology and neurodegeneration

**Fig. 10:**
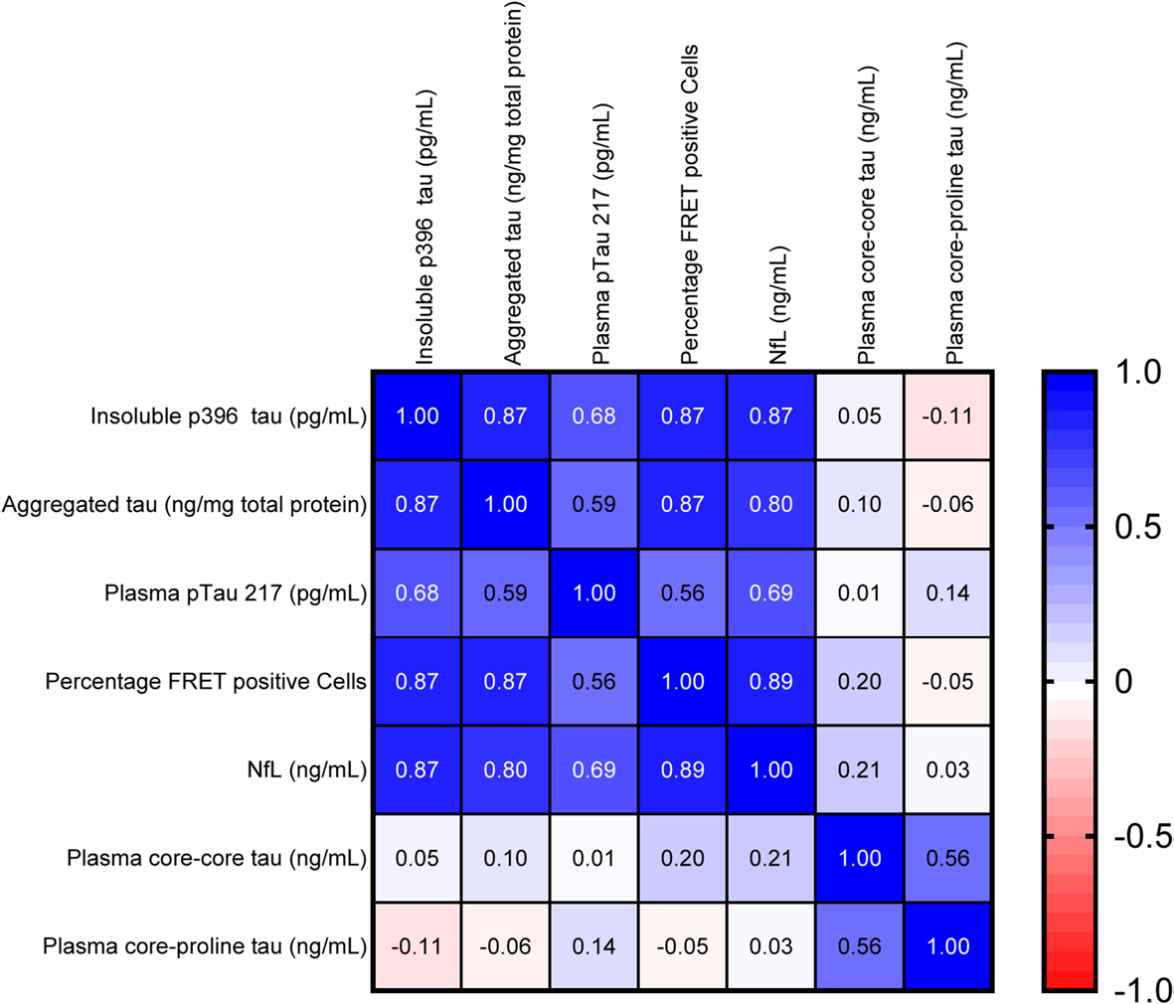
Correlation heatmaps for all measured parameters. Spearman’s rank correlations are displayed in blue for positive correlations, red for negative correlations and white where no correlation was detected (*p* < 0.05)

**Fig. 11:**
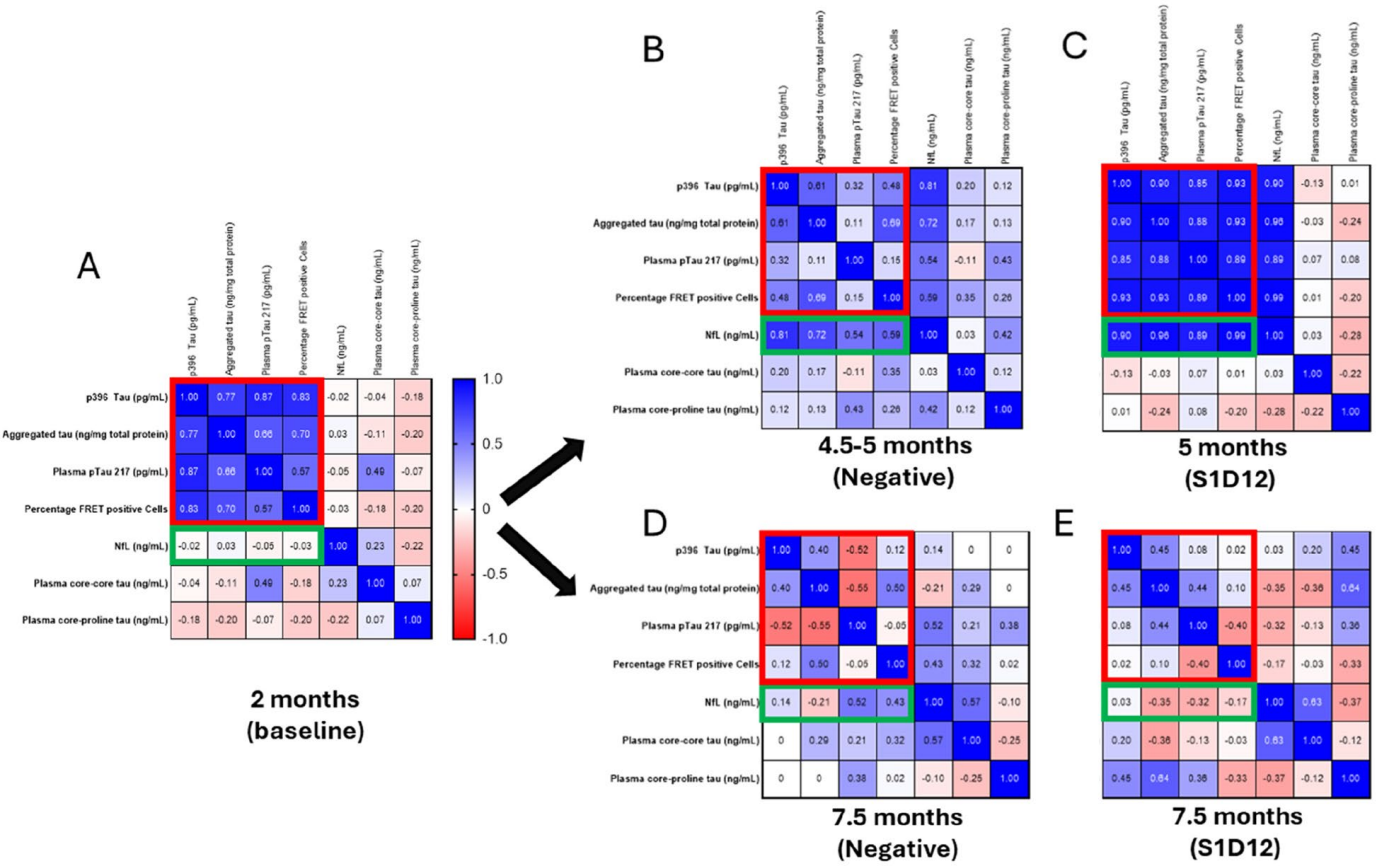
Correlation heatmaps for each age/treatment group. **A** 2-month baseline; **B** 4.5 and 5 month-negative combined; **C** 5-month S1D12; **D** 7.5-month negative and **E** 7.5-month S1D12. Spearman’s rank correlations are displayed in blue for positive correlations, red for negative correlations and white where no correlations were detected

The biochemical endpoints monitoring tau pathology were analysed using Spearman’s rank correlation (Figure S4). Data were converted into heatmaps to visualise all possible correlations in a single figure. When all parameters were considered independent of group (Fig. 10), it appeared that principal parameters for brain (aggregated and insoluble tau), cells (seed competent tau) and plasma (pTau 217 and NfL) correlated highly (see blue tiles with correlation coefficients > 0.59) with each other suggesting that each parameter reflects progression of tau pathology. This was not the case for plasma core-core and proline-core tau, which presented with either weak or no correlation (see whitish tiles with correlation coefficients < 0.22). We continued with a more detailed analysis of each age/treatment group in which the 4.5 and 5 month-negative groups were combined. This correlative pattern was similarly observed in two-month-old mice (Fig. 11A red rectangle); yet there was no correlation with NfL indicating a low level of neurodegeneration at this age (green rectangle). This lack of correlation was confirmed by a 4-parameter logistic regression rectangle (Figure S5). NfL correlations only appeared at negative groups older than 4.5/5 months (Fig. 11B, D, green rectangle). The strength of the correlations for our principal parameters weakened in 4.5/5 and 7.5-month-old negative groups (red rectangles) or even turned negative. Treatment with S1D12 caused differential effects when administered to young or old mice (Fig. 11C, E). In young cohorts, all principal parameters including NfL become highly correlated, potentially due to the strong reduction of all these parameters in this treatment group. Individual two-parameter correlation plots (see Figure S5) confirmed the very low level (dark red circles) of all parameters in this S1D12 cohort. A similar reduction was not observed when S1D12 was administered into older mice with strong pre-established pathology (Fig. 11E, S5). Interestingly, there are only weak or even negative correlations between core-core and core-proline tau with any of the other plasma or brain markers (mostly *p* < 0.5 or *p* > −0.5), particularly in the S1D12 cohorts (Figs. 11C, E). We take this as evidence that the S1D12-dependent increase in target binding is the mechanism by which a lowering of brain and plasma-related tau markers is achieved.

### Summary of in vivo study

The results for S1D12-mediated inhibition of various parameters presented above are summarised in Table 4.

**Table 4:** Summary of S1D12-mediated inhibition of key study and disease-relevant endpoints

Parameter	S1D12-mediated inhibition^a^			
	**Young cohort**		**Old cohort**^**b**^	
	**%**	**p**	**%**	**p**
Aggregated tau	91	**0.0002**	73	0.2766
Seed competent tau	76	**0.0005**	4	0.8473
Insoluble phosphorylated tau (p396)	86	**< 0.0001**	62	**0.0001**
Insoluble phosphorylated tau (p202/205)^c^	97	**< 0.0001**	0	0.8231
Plasma pTau217	55	**0.0233**	48	0.1196
Plasma NfL	60	0.1478	19	0.5126

The overall efficacy of S1D12 is much stronger in young mice compared with older animals (p-values highlighted in bold)

^a^S1D12-mediated inhibition is calculated as relative change from the mean of baseline in S1D12-treated mice when compared to the control negative antibody treatment

^b^The old cohort mice test the ability of S1D12 to remove established tau aggregates rather than inhibiting disease progression

^c^S1D12-mediated inhibition for insoluble phosphorylated tau (p202/205) is calculated using S1D12-treated mice compared to negative control treatment mice and not relative to baseline results

### S1D12 offers potential therapeutic utility in Alzheimer’s disease and other primary tauopathies

In addition to the in vivo study in transgenic tau mice, the therapeutic utility of S1D12 was assessed via immunodepletion of tau seeding species from brain homogenates of histopathologically confirmed cases of various primary tauopathies. The ability of S1D12 to deplete tau seeds in brain homogenates was measured by transfection of Tau RD P301S FRET biosensor cells followed by flow cytometry. A series of different tauopathies was examined: Alzheimer’s disease (AD), behavioural variant frontotemporal dementia (bvFTD), progressive supranuclear palsy (PSP), corticobasal degeneration (CBD) and argyrophilic grain disease (AGD) (see Table 3).

**Fig. 12:**
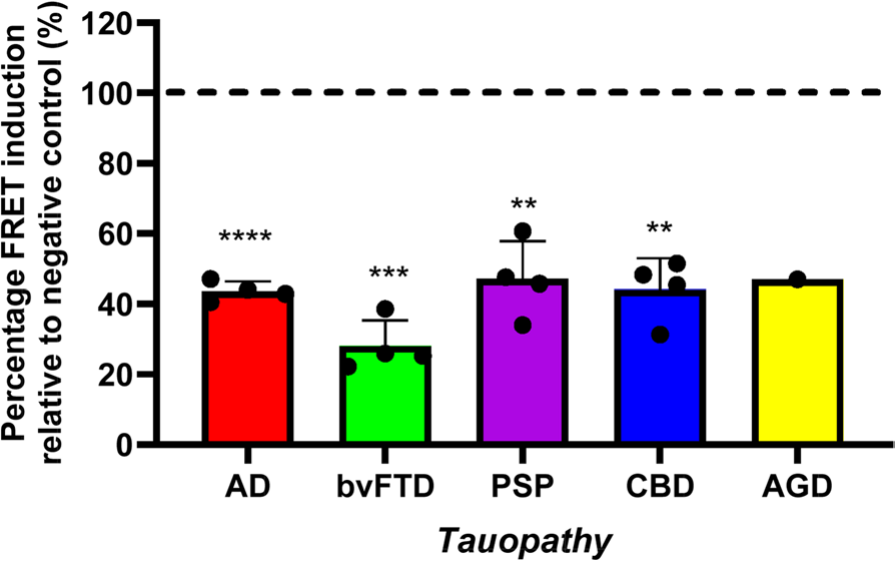
S1D12-mediated inhibition of seeds derived from different tauopathies. Percentage FRET signal generated in Tau RD P301S FRET biosensor cells transfected with brain homogenate (200 µg/mL total protein) following immunodepletion with S1D12 in comparison to a negative control antibody. One-way ANOVA was performed with Tukey’s post hoc test to compare the effect of S1D12 between tauopathies (*F*(4,16) = 4.56: *p* = 0.024). Asterisks indicate comparisons with negative control level (dashed line; **, *p* < 0.01; ***, *p* < 0.001; ****, < 0.0001). AD = Alzheimer’s disease, bvFTD = Behavioural variant frontotemporal dementia, PSP = Progressive supranuclear palsy, CBD = Corticobasal degeneration, AGD = Argyrophilic grain disease. *N* = 4 cases per tauopathy performed in duplicate (except for AGD; *N* = 1), values are expressed as mean + SD and displayed relative to a negative control antibody (Protein A-purified mouse IgG (Immunoreagents, #Mu-003-C.01)

Although the filaments for different tauopathies are structurally diverse [50], S1D12 exhibited relatively universal levels of tau seeding inhibition across all tauopathies tested. FRET induction, relative to the negative control antibody was similar in AD (43.6 ± 2.7%,t = 41.7, df = 3, *p* < 0.0001), PSP (47.0 ± 10.9%, t = 9.7, df = 3, *p* = 0.0023), CBD (44.1 ± 8.9%, t = 12.6, df = 3, *p* = 0.0011), with the highest level of inhibition observed in bvFTD cases (28.0 ± 7.3%, t = 19, df = 3, *p* = 0.0003). There were no statistically significant differences between tauopathies, with the exception of PSP and bvFTD (*p* ≤ 0.05). Although only one case of AGD was available for this analysis, the percentage of FRET induction relative to control (47.0%) was very similar to that for AD, CBD and PSP cases (Fig. 12). S1D12-mediated inhibition of tau seeding was not assessed in healthy control brain samples, as these exhibited no seeding. In short, S1D12 has potential therapeutic utility across a range of tauopathies.

## Discussion

S1D12 is a chimeric IgG2a, consisting of ovine variable regions and murine constant regions. The potential therapeutic utility of S1D12 was investigated in the tau transgenic L66 mouse model to determine whether it can prevent tau aggregation or actively remove existing tau aggregates in vivo or do both.

S1D12 inhibited the temporal progression of tau aggregation without removing existing aggregates. It is likely that this arises as a result of epitopes within the core domain (including the S1D12 epitope) being occluded in the later, filamentous stage of the tau protein [19]. Whilst S1D12 was tested for its ability to remove established tau aggregates in an older cohort of mice, we observed modest progression for some tau endpoints measured across the treatment period. This supports a mechanism for S1D12 halting disease progression and not disease reversal.

It is considered that the active and toxic species of tau protein is predominantly made of oligomers having intermediate solubility, with tombstone ‘tangles’ representing late-stage remnants of neurodegeneration [51, 52]. Soluble tau oligomers are responsible for anterograde trans-synaptic spreading and subsequent loss of brain function [53, 54], further highlighting that targeting these active species of tau with S1D12 rather than tangle removal may prove a more beneficial and efficacious therapeutic strategy against tauopathies.

The premise of arresting the spatiotemporal progression of tau pathology is one of great potential translational benefit to the field of AD and other tauopathies which could offer patients extended years with a retained quality of life. Since tau pathology begins decades before clinical symptoms [55, 56], it would be most advantageous to inhibit this prior to the onset of symptoms and, given the recent advances in fluid and imaging biomarkers for AD, this is becoming increasingly feasible. In particular, recruiting patients early in disease by amyloid imaging, measuring phosphorylated tau fluid biomarkers, thus preventing tau PET positivity or spatiotemporal progression would provide a reasonable rationale to confirm if S1D12 therapy can be used to halt disease progression.

### Comparison with other anti-tau immunotherapies

It is difficult to compare S1D12 to other anti-tau immunotherapies, given the differences between in vivo study designs. These include but are not limited to: administration of exogenous filaments to animals; different tau transgenic animals; different end points chosen; different methods of analysing tau pathology (e.g., immunohistochemistry, biochemical or functional analyses); age of animals; and dosing route and regimen. Despite these caveats, by taking insoluble phosphorylated tau as a commonly used endpoint, S1D12 clearly shows the “best” ability to prevent tau pathology. The most comparable study would be one using semorinemab, an N-terminal tau antibody [57]. This study involved a 13-week treatment (once weekly) of transgenic P301L human tau mice in which no significant differences in insoluble-phosphorylated tau levels were shown between semorinemab-treated and control groups [57]. For comparison, with less frequent dosing, S1D12 inhibited insoluble-phosphorylated tau by up to 97% as measured by AT8 western blotting on brain homogenates. Despite differences in experimental design, it is also worth mentioning E2814 as it is the most closely related antibody to S1D12, because it likewise targets the microtubule-binding repeat (MTBR) region of tau (amino acids 299–303 and 362–366). IP administration of the murine form of E2814, 7G6, was reported to modestly reduce the propagation of insoluble, fibrillar tau from the injected left hemisphere to the contralateral right hemisphere in P301S transgenic mice, following intracerebral administration of tau fibrils [58].

### Target engagement

By utilising Simoa technology, core tau concentrations in mouse plasma were measured which showed that core tau concentrations were increased in the plasma in the range of 1.3 to 6.1-fold (depending on assay) following S1D12 immunotherapy. Not many previous published studies have analysed plasma tau levels, although the semorinemab (epitope tau 6–23) study reported a 15-fold increase in plasma tau after a single i.p. dose of 30 mg/kg in transgenic tau mice [57]. A similar study showed a single i.p. dose (50 mg/kg) of another N-terminal antibody (HJ8.5, epitope tau 25–30) increased plasma tau by 10-fold [59]. Since our analysis was performed after 8 × dosing cycles with S1D12, direct comparison is not appropriate, but the data could be taken as evidence that N-terminal antibodies are causing a larger increase in tau release into blood. Alternatively, the antibodies could be stabilising N-terminal fragments that would normally be cleared and are not affecting the aggregation cascade of tau pathology. Additional plasma assays are required using antibodies that target epitopes outside of the MTBR repeat regions of tau, to confirm that this is not the case in this study. The inverse correlations of core-tau plasma biomarkers with pathological endpoints following S1D12 treatment, however, is suggestive of target engagement (i.e. less tau aggregation in the brain following S1D12 treatment results in higher levels of core-tau in plasma).

### Plasma biomarkers of tau pathology and neurodegeneration

This study was highly biomarker led, and to our knowledge is the only tau therapy in murine models to inhibit the levels in plasma of both pTau217, a surrogate marker for tau pathology, and NfL, a surrogate marker for neurodegeneration arising after the appearance of tau changes.

In the context of AD, there has been discussion around what the presence of pTau biomarkers (including pTau217) in plasma represent [60], they need not necessarily be exclusive surrogate measurements of structural tau pathology. Recent evidence suggests that some pTau biomarkers in blood may in part reflect synaptic dysfunction via accumulation and release of oligomeric phosphorylated tau and also plaque-associated dystrophic neurites that contain oligomeric phosphorylated tau [61–64]. The changes in pTau reported in our study, however, are not amyloid induced given that the mouse model is solely transgenic for tau. L66 mice express full-length human tau carrying the P301S mutation that is associated with FTD and they resemble FTD both in the pattern of neurodegeneration and phenotypically. L66 mice show aggressive early-onset neurofibrillary degeneration, and their behavioural phenotype resides largely in the motor function domain [20]. Despite the similarities with FTD, the increased plasma pTau217 in L66 is not consistent with the absence of raised pTau217 in FTD subjects [42]. The fact that S1D12 appears to reduce the level of plasma pTau217 (that also correlates with brain pathology) suggests that changes in disease-relevant tau are being inhibited by S1D12 and that pTau217 is a surrogate measure of disease modification.

Although decreased plasma NfL by S1D12 did not reach statistical significance (*p* = 0.1478), this is not surprising given the small number of mice and biological variance of the data. Nevertheless, the large effect size (60% inhibition) in the young mice is encouraging and the finding is further supported by the highly significant correlations between NfL and tau pathology endpoints.

### Relevance of S1D12 to various primary tauopathies

The tau filament fold, comprising of three repeat (3R) and/or four repeat (4R) tau isoforms, differs in various tauopathies as determined by cryo-EM [50]. For example, 3R tau isoforms are found in filaments of Pick’s disease, whereas 4R tau isoforms are present in PSP, CBD and AGD. In AD, filaments contain a mixture of 3R and 4R isoforms. However, the tau folds for AD and all of the primary tauopathies have a common core region that incorporates the MTBR repeat regions R3 and R4 [65]. Since S1D12 binds to R4, it was hypothesised that it could also target tau seeding species in other primary tauopathies, potentially offering therapeutic benefits across these diseases. This was confirmed by the ability of S1D12 to bind and deplete tau seeds in brain homogenates of the diverse primary tauopathies. Here, we show that S1D12 exhibits a relatively universal level of tau seeding inhibition for AD, bvFTD, PSP, CBD and AGD.

### Further experiments

Further exploratory work is required to analyse immune and pro-inflammatory biomarkers in the brain and periphery of S1D12-treated animals compared to controls. This is of relevance given that pathological tau induces microglial activation and inflammatory markers [66] and it will be of interest to see if S1D12 can neutralise or minimise such inflammation and its triggers in vivo. Furthermore, different isotypes of S1D12 could be tested since it remains unclear which IgG isotype would be best for tau immunotherapy and whether microglial engagement is necessary and appropriate [67–69].

Although the tau-based endpoints measured within this study were closely correlated, there is evidence within this study that they may be measuring related but not identical pathological tau species and this warrants further investigation. For example, AT8 western blots showed no difference between 4.5-month baseline and 7.5-month, whereas plasma pTau217 showed a 3.2-fold change over this treatment period. Plasma pTau217 may reflect synaptic dysfunction and dystrophic neurites releasing soluble oligomeric phosphorylated tau [63, 64] whereas AT8 detects structural, filamentous tau pathology [39].

### Other considerations particular to the in vivo model used

This ‘proof-of-concept’ study was only carried out in female mice which is acceptable for this context but should be further investigated in males.

Although the P301S tau mutation used in the L66 model is associated with FTD [70], mutations in tau are not associated with AD. The L66 model shows behavioural characteristics similar to FTD and exhibits tau aggregation [20]. Further studies in other mouse models may better reflect AD. Furthermore, to more clearly define the therapeutic effects of S1D12, future studies should incorporate behavioural assessments alongside histological analyses to evaluate the distribution and extent of tau pathology.

## Conclusion

S1D12 inhibits the progression of tau pathology in tau transgenic mice. Anti-core tau immunotherapy as a mechanism of action seems to be exclusive to the inhibition of aggregation rather than removal of established pathology and this should be considered within any future clinical trial design. This study supports the hypothesis that starting immunotherapies, for a range of tauopathies, early and even prior to symptoms could significantly improve long term efficacy.

## Supplementary Information

Supplementary Material 1.

## Data Availability

All data are provided within the manuscript or the Supplementary figures/tables. Data and materials are available from corresponding author, Mohammad Arastoo, on reasonable request.

## Abbreviations

2N4R tau: Full-length tau (1–441 AA)
4PL: Four-parameter logistic regression
AA: Amino acid
AD: Alzheimer’s disease
ANOVA: Analysis of variance
Aβ: Amyloid beta protein
BCA: Bicinchoninic acid (assay)
BSA: Bovine serum albumin
CFP: Cyan fluorescent protein
C_max_: Maximum observed concentration
CSF: Cerebrospinal fluid
dGA: Tau297-390 fragment of 2N4R tau
dGAE: Tau297-391 fragment of 2N4R tau
DMEM: Dulbecco’s modified eagle medium
EDTA: Ethylenediaminetetraacetic acid
ELISA: Enzyme-linked immunosorbent assay
FDA: Food and drug administration
FRET: Fluorescence resonance energy transfer
FTD: Frontotemporal dementia
HEK: Human embryonic kidney
HRP: Horseradish peroxidase
i.p.: Intraperitoneal
IgG: Immunoglobulin G
IHC: Immunohistochemistry
ISF: Interstitial fluid
L1: Line 1 mice
L66: Line 66 mice
Lambda_z: Terminal elimination rate constant
mAb: Monoclonal antibody
MTBR: Microtubule-binding region
NfL: Neurofilament light chain
NMRI: Naval Medical Research Institute (mice)
p202/205: Tau phosphorylated at Ser-202 and Thr-205
p396: Tau phosphorylated at Ser-396
PBS: Phosphate-buffered saline
PBST: Phosphate-buffered saline plus Tween® 20 (0.1%)
PET: Positron emission tomography
PHF: Paired helical filament
PK: Pharmacokinetic
pTau: Phosphorylated tau
RGP: Resorufin β-D-galactopyranoside
RIPA: Radioimmunoprecipitation assay (buffer)
SBG: Streptavidin β-galactosidase
SDS-PAGE: Sodium dodecyl-sulfate polyacrylamide gel electrophoresis
SEM: Standard error of the mean
Simoa: Single molecule array
t_1/2_: Terminal half-life
TBS: Tris-buffered saline
TBST: Tris-buffered saline plus Tween® 20 (0.1%)
T_max_: Time point of maximum observed concentration
TMB: 3,3′,5,5′-Tetramethylbenzidine
WB: Western blot
YFP: Yellow fluorescent protein
