# Supplementary material for: Timing matters: early administration of a high-affinity antibody targeting the tau repeat domain prevents aggregation in a mouse tauopathy model
Source: Alzheimers Res Ther. 2026 Feb 14;18:65. doi: 10.1186/s13195-026-01985-x (PMC13020213; doi:10.1186/s13195-026-01985-x)
Supplement: Supplementary file 1 — Supplementary Material 1. [file 13195_2026_1985_MOESM1_ESM.docx]

**Timing matters: early administration of a high-affinity antibody targeting the tau repeat domain prevents aggregation in a mouse tauopathy model**

**Lewis K. Penny ^,1,2,4^, Mohammad Arastoo ^1,2^, Richard Lofthouse ^1,2^, Aya Abdallah ^1,2^, Peter I. Imoesi ^1^, Karima Schwab ^1^, Helen Shiells ^4^, Valeria Melis ^1^, Gernot Riedel ^1^, Charles R. Harrington ^1,3,4^, Claude M. Wischik ^1,3,4^, Andrew Porter ^1,2^, Soumya Palliyil ^1,2^.**

^1^ Institute of Medical Sciences, University of Aberdeen, Aberdeen, UK

^2^ Scottish Biologics Facility, University of Aberdeen, Aberdeen, UK

^3^ GT Diagnostics (UK) Ltd., Aberdeen, UK

^4^  TauRx Therapeutics Management Ltd., Aberdeen, UK

^#^ Correspondence: [mohammad.arastoo@abdn.ac.uk](mailto:mohammad.arastoo@abdn.ac.uk)

**Supplementary Materials**

**Supplementary Tables – 2 Tables**

**Supplementary Figures – 3 Figures**

**Supplementary tables**

Table S1: Number (n) of mice used in the 12-week repeat dose study. Animals excluded from analysis due to non-treatment related welfare reason are indicated in parentheses. Additionally, one brain in group 1a and one in group 2a were not collected.

**Group 2 (n)**

**2 month (Baseline)**

**5 month (Negative)**

**5 month (S1D12)**

**4.5 month (Baseline)**

**7.5 month (Negative)**

**7.5 month (S1D12)**

**Brain**

**9**

**10**

**10**

**9**

**10**

**10**

**Plasma**

**10**

**10**

**10**

**10**

**8**

**10**

**Aggregated tau**

**9**

**10**

**10**

**9**

**8**

**10**

**Seed competent tau**

**9**

**9**

**10**

**9**

**8**

**9**

**insoluble p396 tau (pg/mL)**

**9**

**10**

**10**

**9**

**8**

**10**

**pTau217 (pg/mL)**

**9**

**10**

**10**

**10**

**8**

**10**

**NFL (ng/mL)**

**10**

**10**

**10**

**10**

**8**

**10**

**Plasma core-proline tau (ng/mL)**

**10**

**9**

**10**

**10**

**8**

**10**

**Plasma core-core tau (ng/mL)**

**7**

**10**

**10**

**10**

**7**

**10**

**Group 1 (n)**

**Table S2. Number (n) of samples used in this study.** Samples collected at the end of the 12-week repeat-dose study are highlighted in red, while those used for individual assays are shown in blue. In some experiments, data points are missing due to factors such as sample unavailability, assay error messages, or signal levels below the assay blank.

**Supplementary figures**

**Effect of treatment on body weight**

The body weight of mice in groups 1 and 2 recorded before the dosing phase is shown in Figure 1. A significant difference was detected, with 4.5-month-old presenting with higher body weight than the younger cohort (Mann-Whitney test: p=0.002).

To investigate the effect of treatment on animal body weight, the percent weight change was recorded at the end of the study with respect to the initial weight (Fig. S2). One sample t test was used to compare the mean of each group with a hypothetical value of zero corresponding to “no-variation” level. L66 mice in group 1 (5-month-old at tissue harvesting) gained significant body weight at the end of the 12-week dosing phase (p values < 0.0001 from theoretical value zero) whereas older mice in group 2 (7.5-month-old at tissue harvesting) did not show any significant difference (p values > 0.057 from theoretical value zero).

Within each age-group, no differences were detected between S1D12- and control-treated mice, indicating no-treatment dependent effects on the body weight.

***Figure S1: Body weight recorded at the beginning of the treatment phase for groups 1 (2-month-old) and 2 (4.5-month-old).*** Results are expressed as mean value (± SD). A significant difference was detected for 4.5-month-old presenting with higher body weight than younger cohort (Mann-Whitney test: p=0.002).

Figure S2: Change in percentage body weight after 12 weeks treatment with control or S1D12. One sample t test was used to compare the mean of each group with a hypothetical value of zero corresponding to “no-variation” level. Data are expressed as mean percent body weight change (± SD) at the end of the study with respect to initial weight. #, significant increase in body weight.


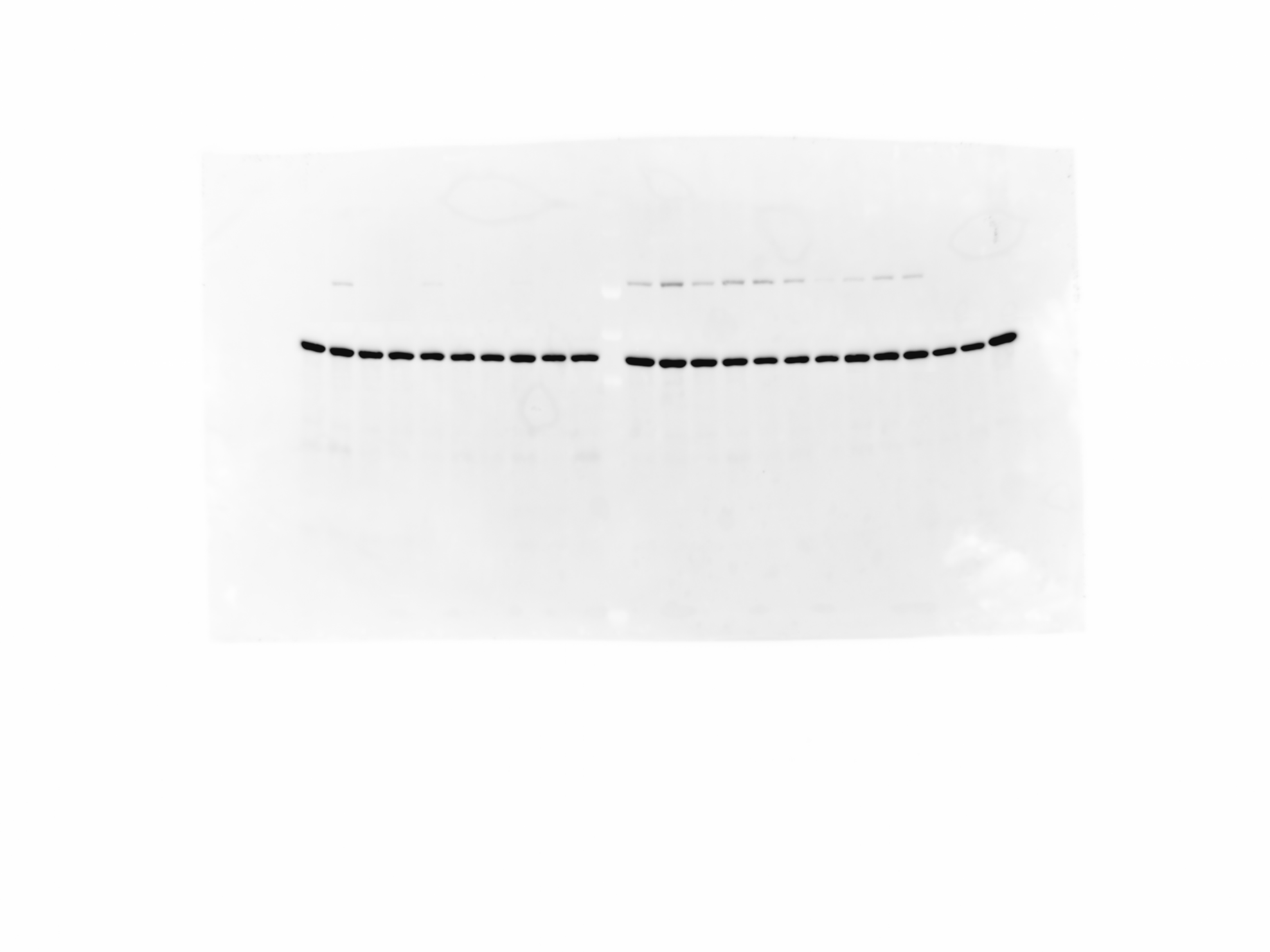


**Figure S3:** Uncropped Western blot of 5-month-old L66 mice brain homogenates shown in Figure 6B, where p202/205 was detected using AT8 antibody (1:500) and beta-actin was detected using AC-74 antibody (1:5000). The first 3 lanes are internal negative controls. Lane 1= WT mouse brain homogenate, lanes 2 and 3= Line 1 transgenic mice brain homogenates.

**
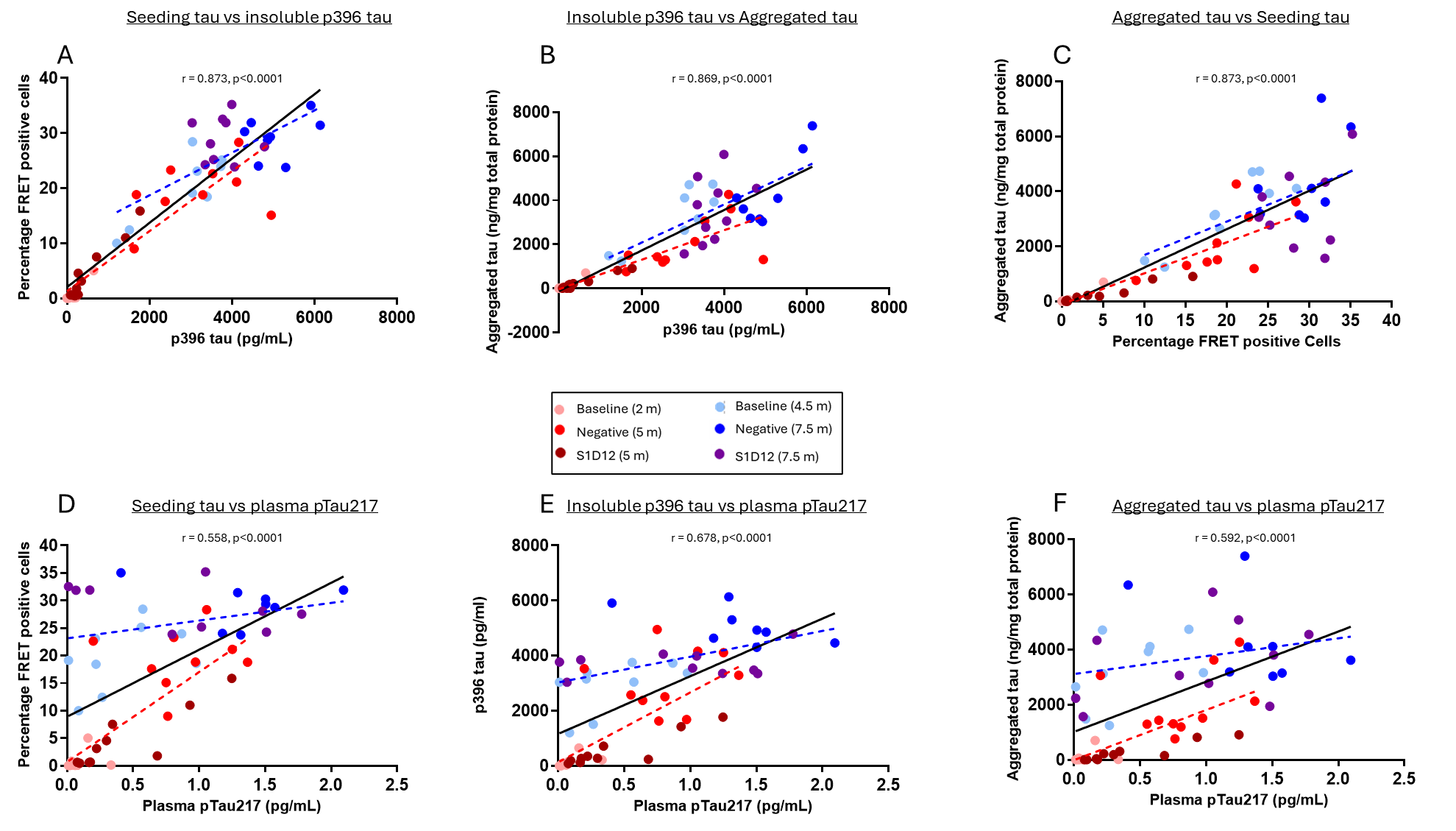
**

**Figure S4: Correlation and linear regression analyses for six different measures of tau pathology.** Linear regression (black line), Spearman correlation coefficients (r) and P values are shown for overall-group analysis. Linear regression is also shown for young (baseline 2-month, negative 5-month and S1D12 5-months; red dashed line) and old (baseline 4.5-month, negative 7.5-month and S1D12 7.5-months; blue dashed line) groups analysed separately.


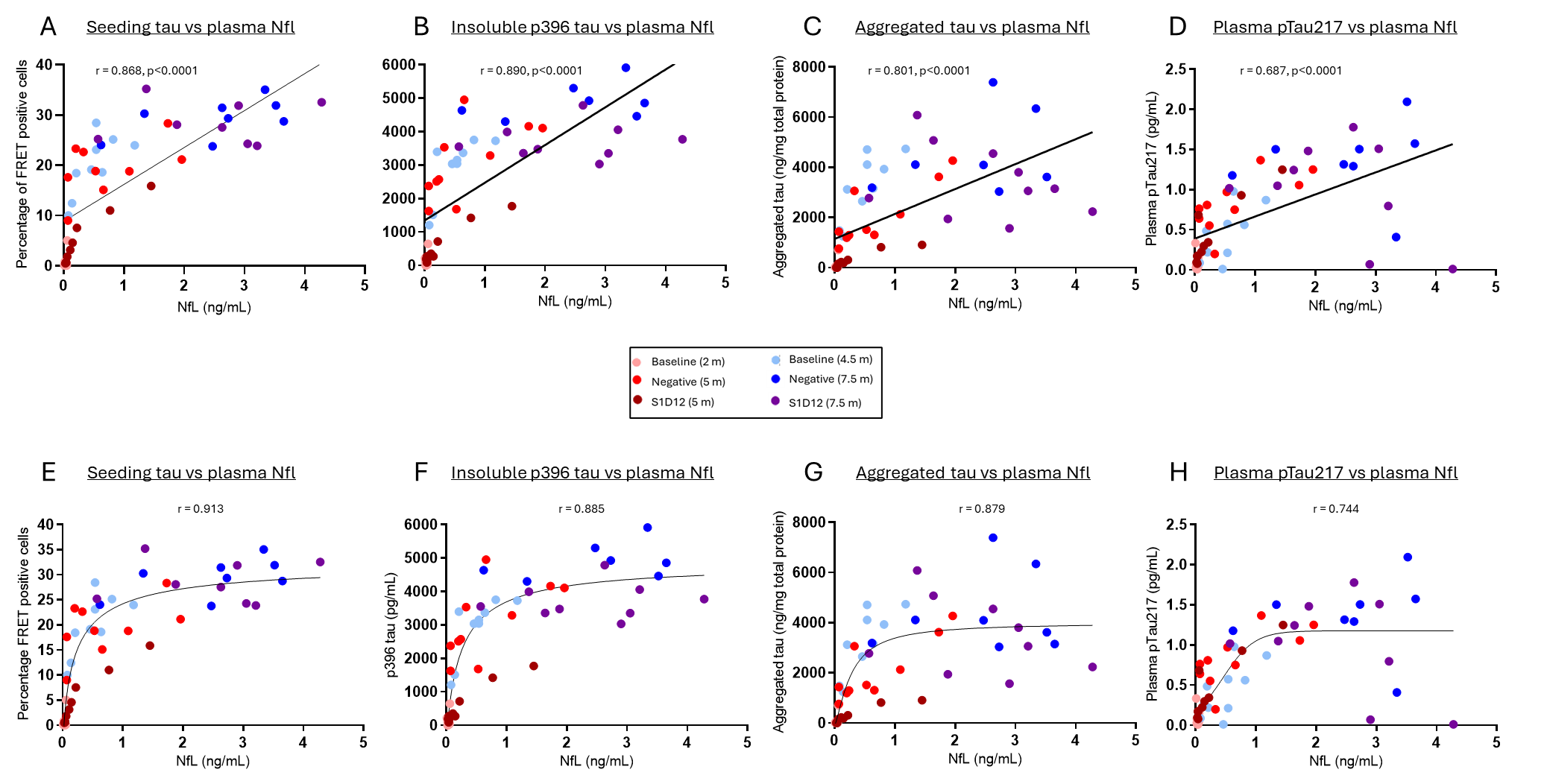


**Figure S5: Correlation, linear regression (A-D) and 4 parameter logistic regression analyses (E-H) for measure of tau pathology and plasma NfL.**
